# Understanding the Complex Surface Interplay for Extraction: A Molecular Dynamics Study

**DOI:** 10.1002/chem.202002744

**Published:** 2020-10-14

**Authors:** Roberto Macchieraldo, Johannes Ingenmey, Barbara Kirchner

**Affiliations:** ^1^ Mulliken Center for Theoretical Chemistry Rheinische Friedrich-Wilhelms-Universität Bonn Beringstrasse 4+6 53115 Bonn Germany

**Keywords:** interfaces, liquid–liquid extraction, molecular dynamics, phase separation, solvometallurgy

## Abstract

By means of classical molecular dynamics simulation the interfacial properties of methanol and *n*‐dodecane, which are two potential candidate solvents for use in non‐aqueous liquid–liquid extraction, were assessed. The question of how the interface changes depending on the concentration of extractant (tri‐*n*‐butyl phosphate) and salt (LiCl) is addressed. Two different models to represent systems were used to evaluate how LiCl and tri‐*n*‐butyl phosphate affect mutual miscibility, and how the last‐named behaves depending on the chemical environment. Tri‐*n*‐butyl phosphate increases the mutual solubility of the solvents, whereas LiCl counteracts it. The extractant was found to be mostly adsorbed on the interface between the solvents, and therefore the structural features of the adsorption were investigated. Adsorption of tri‐*n*‐butyl phosphate changes depending on its concentration and the presence of LiCl. It exhibits a preferential orientation in which the butyl chains point at the *n*‐dodecane phase and the phosphate group points at the methanol phase. For high concentrations of tri‐*n*‐butyl phosphate, its molecular orientation is preserved by diffusion of the excess molecules into both the methanol and *n*‐dodecane phases. However, LiCl hinders the diffusion into the methanol phase, and thus increases the concentration of tri‐*n*‐butyl phosphate at the interface and forces a rearrangement with subsequent loss of orientation.

## Graphical abstract

**At the interface**: The interfacial properties of methanol and *n*‐dodecane, which are candidate solvents for nonaqueous liquid–liquid extraction, were assessed by molecular dynamics simulation. Two different models were used to evaluate how an extractant (tri‐*n*‐butyl phosphate) and a salt (LiCl) affect mutual miscibility, and how the last‐named behaves depending on the chemical environment. Tri‐*n*‐butyl phosphate increases the mutual solubility of the solvents, whereas LiCl counteracts it.

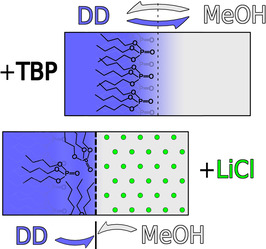

## Introduction

Hydrometallurgy involving aqueous chemistry is today the most common approach for the recovery of metals, and though it will remain a fundamental tool for the extraction of many elements, new routes should be explored. In fact, hydrometallurgy usually involves a combination with pyrometallurgy,[^1^, ^2^, ^3^] which was proven to be insufficient for treating low‐grade ores or residues in an economic way and are weakly selective. The aforementioned reasons, together with the objective to establish a circular economy,^[4]^ led to the development of the innovative concept of solvometallurgy.^[5]^

The idea of solvometallurgy is to exploit processes similar to those of hydrometallurgy but without an aqueous phase. Here, the term “nonaqueous solvent” is used in an inappropriate manner, since it does not necessarily imply anhydrous conditions, but rather a solvent in which the water content is lower than 50 vol %. This opens a broad range of solvents to choose from, including molecular organic solvents, ionic liquids, deep‐eutectic solvents, and inorganic solvents.[^6^, ^7^, ^8^, ^9^, ^10^, ^11^, ^12^]

Some examples of solvometallurgical processes are the recovery of copper from chrysocolla,[^13^, ^14^] rare earths and other metals from complex silica‐rich ores,^[15]^ uranium from carbonate ores, and reprocessing of spent nuclear fuel.^[16]^ As in hydrometallurgy, also in solvometallurgy conventional solvent extraction is used. In this case though, the aqueous phase is replaced by a nonaqueous solvent.[^17^, ^18^]

Although these processes can be carried out easily on a laboratory scale, on an industrial scale they may be challenging and several conditions should be fulfilled.[^5^, ^19^] Among those, in solvent extraction the transition region between the immiscible liquid phases is of paramount importance, as it can either facilitate or hinder the migration of the target compound between the phases and even increase or decrease the selectivity. For these reasons, over the years several studies have been undertaken to characterize the interface between solvents for classical solvent extraction. For example Wipff et al. studied and characterized the interface between aqueous phases and different organic compounds, also in the presence of acids or extractants.[^20^, ^21^, ^22^, ^23^] They proposed an extraction mechanism that involves the adsorption of the ligand at the interface followed by a series of complexation equilibria between ligands and extraction targets resulting in desorption of the adsorbed complex into the organic phase. They also pointed out that desorption of the complex might be facilitated by an increased interfacial concentration of the complex, the extractant molecules, or any other surfactant, which would reduce the interfacial pressure.^[21]^ Moreover, since the complexation of the target by the ligand seems to occur at the interface, the study of ligand affinity toward specific targets (e.g., by quantum chemical methods) should not neglect the impact of the interface structure, and therefore a deeper knowledge of the structural features of the interface is mandatory.

Most of the previous studies were focused on providing fundamental insight into designing aqueous solvent extraction. Obviously, in view of the emerging concept of solvometallurgy, these principles should be expanded to a broader set of immiscible pairs of solvents. Previously, we investigated the mixture of methanol (MeOH) and *n*‐dodecane (DD) in order to discuss phase separation, and studied how the system can be altered and the leading principles for optimizing the system.^[19]^ In the present work, we employed the same mixture to characterize the interface between two immiscible molecular solvents by means of molecular dynamics simulations. We assess at a molecular level to what extent the solvents form distinct phases and study the structural features of the interface between them. In order to do so, we also added a surface‐active ligand, namely tri‐*n*‐butyl phosphate (TBP), and a salt, namely LiCl, which was shown to improve the phase separation of these solvents, to the system. This is a first step towards the understanding of how the interface structure can influence solvent extraction processes and therefore of how these processes can be improved.

## Experimental Section

### Investigated systems

**Figure 1:**
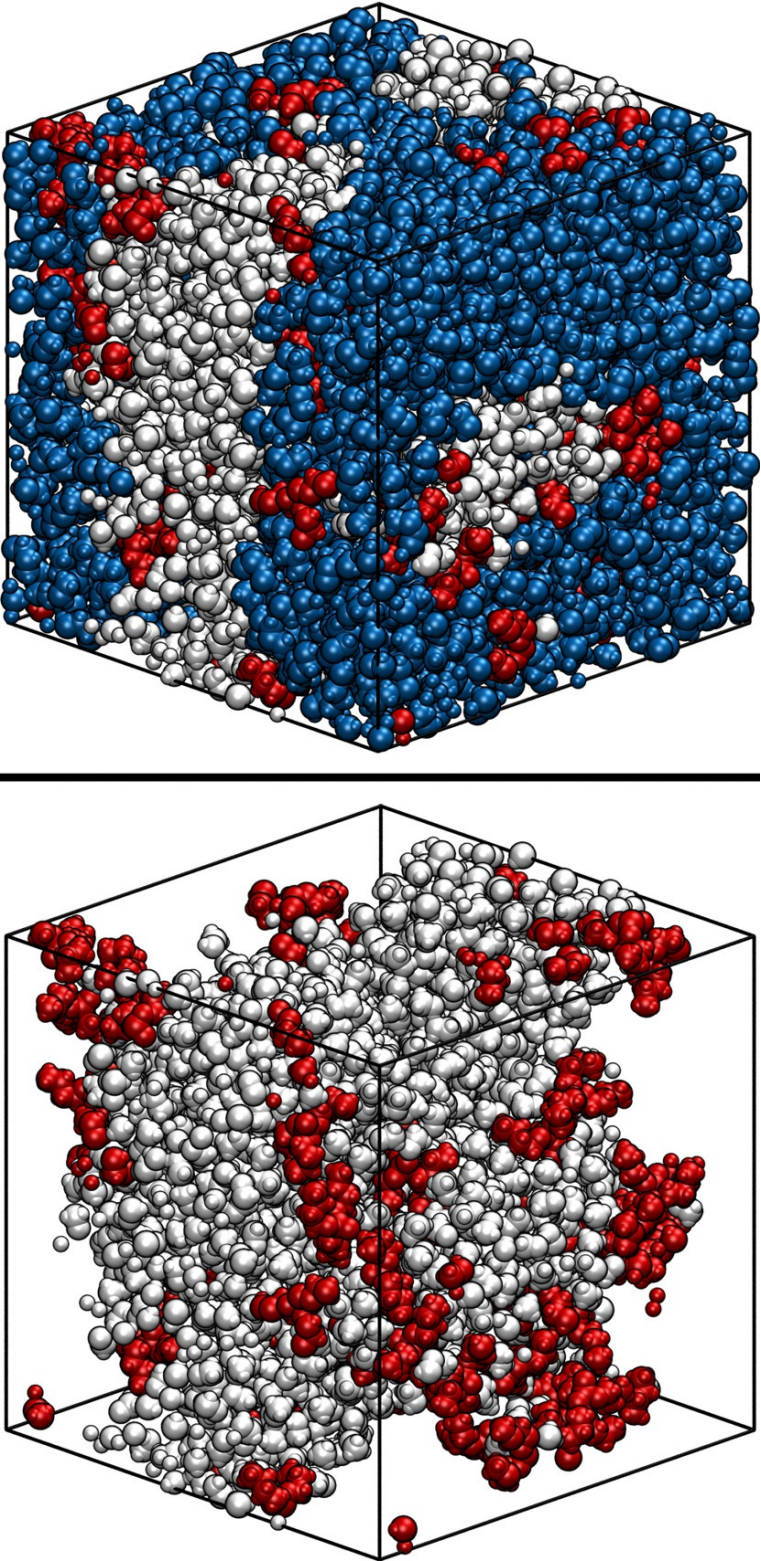
Snapshots of the CUB‐MDT60 system. Atoms are represented by van der Waals spheres. White: MeOH, blue: DD, red: TBP. Top panel: full system, bottom panel: MeOH and TBP only.

**Figure 2:**
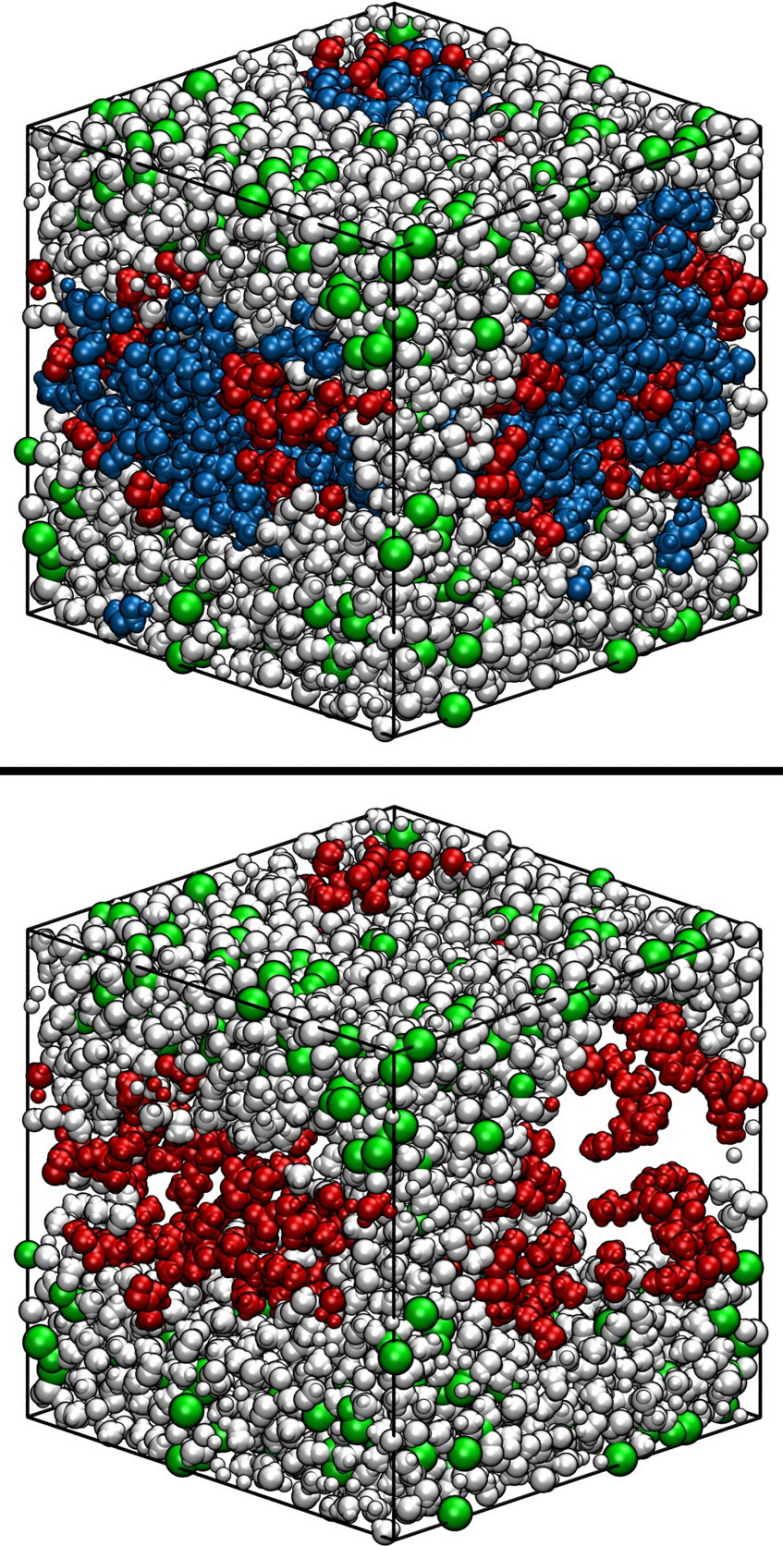
Snapshots of the CUB‐MLDT60 system. Atoms are represented by van der Waals spheres. White: MeOH, blue: DD, red: TBP, green: LiCl. Top panel: full system, bottom panel: MeOH, LiCl and TBP only.

**Figure 3:**
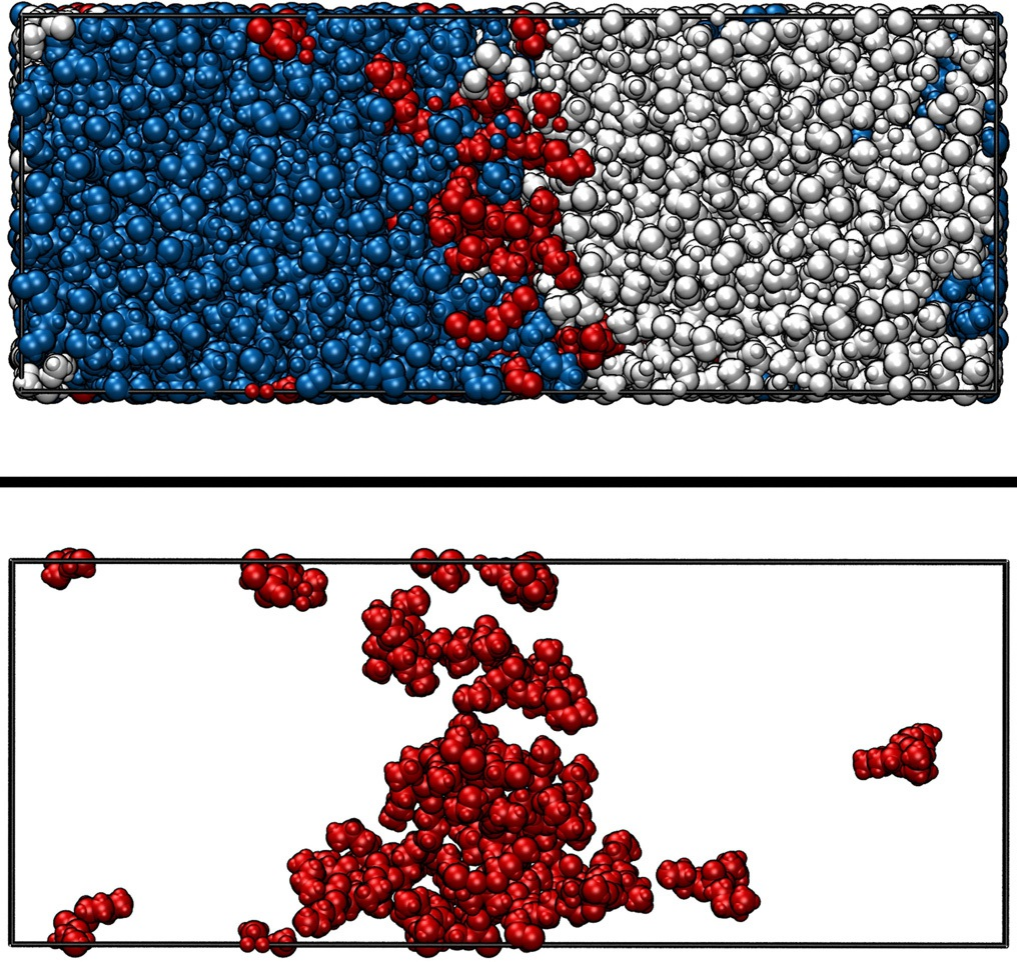
Snapshots of the NCUB‐MDT30 system. Atoms are represented by van der Waals spheres. White: MeOH, blue: DD, red: TBP. Top panel: full system, bottom panel: MeOH, LiCl and TBP only.

We simulated 15 systems, which can be divided into two categories. The first category contains eight biphasic systems simulated in cubic boxes (Figures 1 and 2). These systems were fundamental first to assess how well the force field could represent the phase separation, and second to analyze the mutual miscibility of the solvents at different ligand and salt concentrations. Therefore, their initial configurations were randomly generated to ensure that the obtained phase separation and the interface between the formed phases were not conditioned by the starting geometry or other constraints. The second category contains seven systems, for which the simulation box is longer in the *z* direction (Figure 3). These simulations allow us to investigate interfacial structure and the behavior of the components adsorbed on the interface (technically, two interfaces are generated because of periodic boundaries conditions, but in order to have control over the analyses, TBP was placed only at one interface). Different from the first category, in which the cubic simulation boxes were packed with a random configuration, systems belonging to the second category were generated by juxtaposing the two phases, in order to facilitate equilibration. Thus, the generated interfaces are flat and the behavior of the adsorbed molecules can be easily studied.

The first and second categories of systems are labeled with the initial strings CUB‐ and NCUB‐ respectively. In both cases the remaining letters of the labels represent the components of the system, that is, M stands for MeOH, L for LiCl, D for DD, and T for TBP. More details of the compositions of the systems are listed in Table 1.

**Table 1:** Compositions of simulated biphasic systems. Data refer to the number of molecules in each system.

System/molecules	MeOH	LiCl	DD	TBP
Category 1				
CUB‐MD	1800	–	300	–
CUB‐MDT15/30/60	1800	–	300	15/30/60
CUB‐MLD	1800	217	300	–
CUB‐MLDT15/30/60	1800	217	300	15/30/60
Category 2				
NCUB‐MD	1800	–	300	‐
NCUB‐MDT15/30/60	1800	–	300	15/30/60
NCUB‐MLDT15/30/60	1800	217	300	15/30/60

### Computational details

The initial configurations of the systems were generated by using the PACKMOL package (version 17.039).^[24]^ An initial cell vector of 6.4–6.7 nm was used for the CUB systems, depending on the number of molecules in the cell. Molecules were randomly placed within these boundaries. For the NCUB systems, MeOH and DD were placed in equally sized opposing cells of 6.0 nm length in *x* and *y* directions and 4.0–4.3 nm length in the *z* direction. TBP was randomly placed within 0.5–1.1 nm distance to the interface between the two phases, depending on the number of TBP molecules. Li^+^ and Cl^−^ were placed randomly and independent from each other within the MeOH phase. Classical molecular dynamics was performed by using the LAMMPS program package (version 17 Nov 2016).^[25]^ The well‐known OPLS‐AA force field was used for MeOH, DD, and LiCl,[^26^, ^27^] whereas for TBP we opted for the force field recently developed by Ali et al.^[28]^ since it was shown to perform very well when TBP is mixed with *n*‐dodecane. Nonbonded interactions were described by the 6–12 Lennard–Jones potential and Lorentz–Berthelot mixing rules for pairs of different atoms.^[29]^ A cutoff of 1.6 nm was selected for the calculation of Lennard–Jones and Coulombic interactions together with a particle–particle particle‐mesh solver mapping the atom charge to a 3D mesh.^[30]^ Equilibration of the systems was obtained by simulating for 2.3 ns with *NVE*, *NpT*, and *NVT* ensembles. After an initial energy minimization, the systems were simulated for 0.3 ns in an *NVE* ensemble with added velocity scaling, during which the simulation box was deformed so as to reach a pre‐equilibration density of 0.8 g cm^−3^. Following this, the systems were simulated for 0.5 ns in an *NpT* ensemble by applying the Nosé–Hoover chain thermostats and barostat to achieve constant pressure and temperature (*T=*297.15 K, *τ*=100 fs and *p=*1 bar, *τ*=1000 fs, respectively).[^31^, ^32^] Finally, using the average volume of the previous 0.05 ns as final box volume, the system was equilibrated for another 1.5 ns in an *NVT* ensemble with the same settings for the thermostat. The subsequent production run consisted of 10 ns of simulation time in an *NVT* ensemble, with the same settings as during equilibration. The time step was set to 0.5 fs during the whole procedure, and every 1000th time step was saved in a trajectory for further processing.

### Computational analyses

The obtained trajectories were analyzed with the TRAVIS software and other in‐house scripts.[^33^, ^34^] TRAVIS offers different kinds of functions allowing the analysis of the interaction among the components of the systems. Intra‐ and intermolecular interactions can be taken into account.

The domain analysis feature implemented in TRAVIS, which is based on Voronoi tessellation, was employed to study the phase separation of the solvents and mutual miscibility.^[35]^ Radical Voronoi tessellation[^35^, ^36^, ^37^] was used on every saved trajectory time step to calculate the number of molecules that migrated from one phase to the other, and thereby to study how mutual miscibility changes in different conditions. The Voronoi tessellation works as follows: the system is divided into subsets, wherein a subset is a set of specific atoms. Subsets can either contain all atoms belonging to a molecule or atoms selected from different molecules depending on the aim of the analysis. Once the atoms are flagged for the subset they belong to, the algorithm analyzes the interconnection of the atoms belonging to the same subsets. Atoms of the same subset that are in close contact with each other are considered part of the same domain, and the total number of domains formed by each subset describes how the subset is distributed in the system. When the subset contains all atoms of a specific compound, a number of domains equal to unity means that this compound tends to aggregate. On the other hand, a number of domains equal to the number of molecules of the compound means that the compound is fully solvated by another solvent. Using radical Voronoi tessellation, Brehm and Sebastiani were able to successfully determine the exposed surface of 1‐ethyl‐3‐methylimidazolium acetate droplets in vacuum and used it to describe a liquid–vacuum interface.^[38]^ In this work, which treats a liquid–liquid interface, raw data from the Voronoi tessellation analysis were processed to obtain information on the number of molecules of one solvent or compound dissolved into the other. This was possible because of the neat phase separation of the two solvents. Phase separation generates two main domains that contain most of the atoms belonging to the subset. Therefore, by counting the number of atoms of the subset that do not belong to the main domain, it is possible to calculate the number of molecules that migrated into the other solvent. For the Voronoi tessellation‐based analyses in this work, MeOH and DD were treated as separate subsets, while TBP and LiCl were included as Voronoi sites but were not analyzed further.

**Figure 4:**
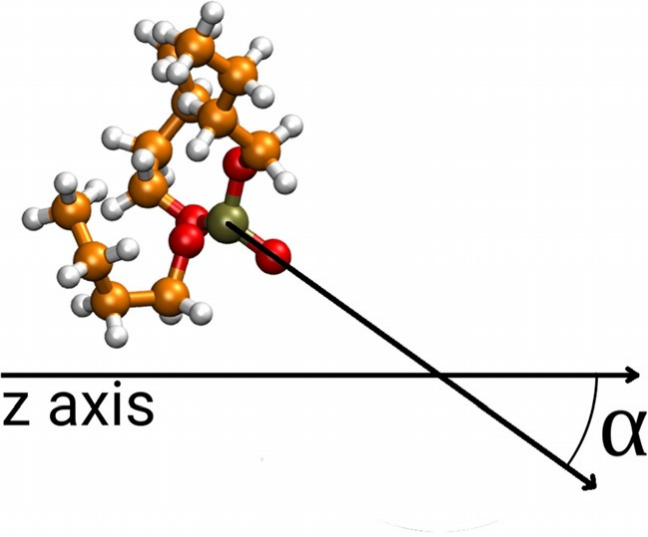
Angle *α*, that is used to assess the orientation of TBP.

Combined distribution functions were generated by correlating the positions of TBP molecules with their orientation and by applying the Kernel Density Estimation provided in the Python library Seaborn.^[39]^ The orientation of the molecules was evaluated by the angle *α* between the *z* axis and the vector whose base and tip are positioned on P and O of the P=O bond, respectively, as shown in Figure 4. The 1/sin *α* cone correction to correct the uniform angular distribution was applied.^[33]^

## Results

### Mutual dissolution of solvents

**Figure 5:**
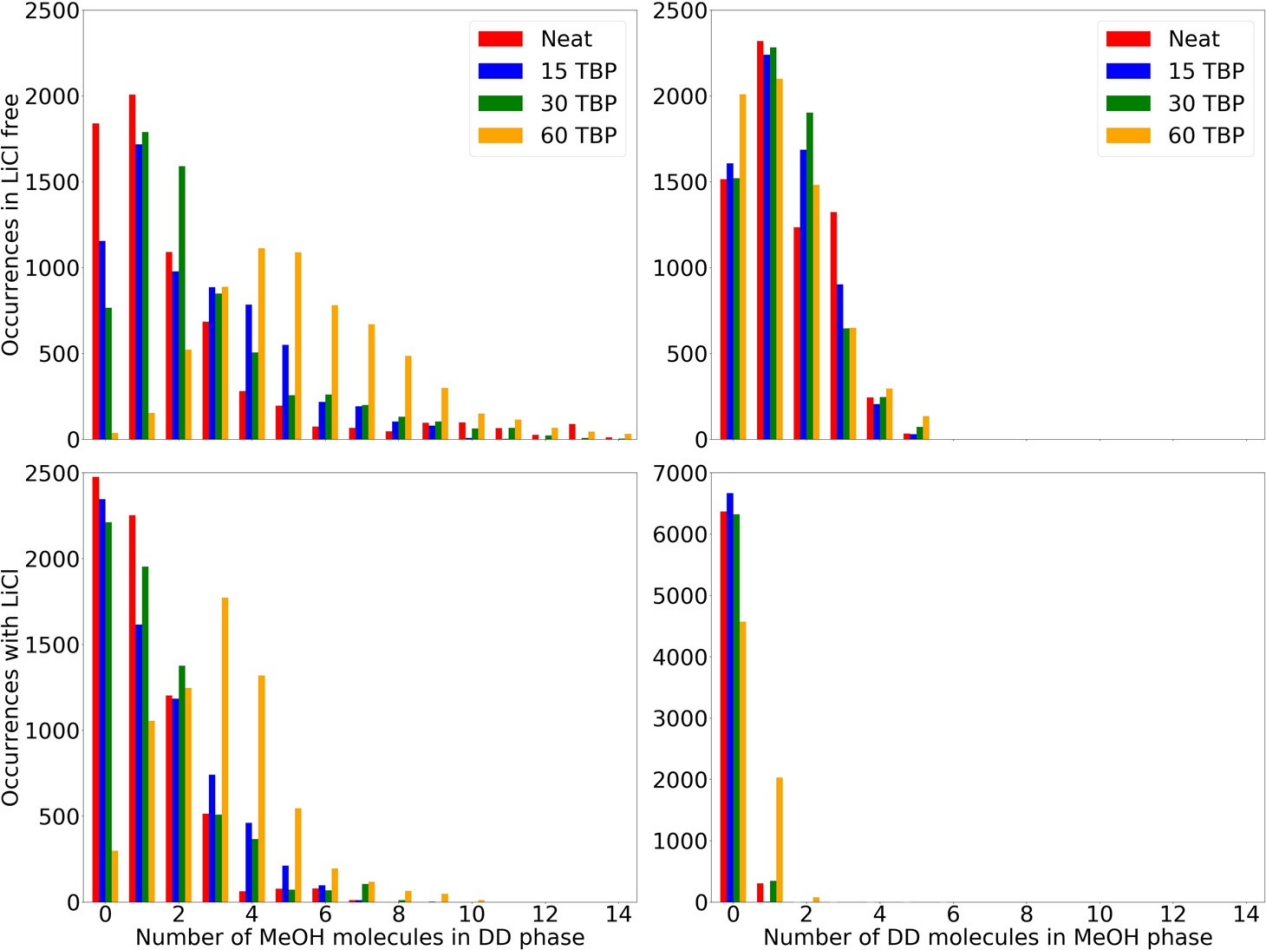
Left: Number of MeOH molecules migrated into the DD phase over the simulation time. Colors represent different TBP concentrations. The top panel shows the distribution in the LiCl‐free system, and the bottom panel that in systems with LiCl. Right: Number of DD molecules migrated into MeOH phase over the simulation time. Colors represent different TBP concentrations. The top panel shows the distribution in the LiCl‐free system, and the bottom panel that in systems with LiCl.

The distribution of the components in the different formed phases was analyzed with the CUB systems. We used Voronoi tessellation (see Methods Section) to calculate the number of molecules that migrated from one phase to the other in each time step. The results are summarized as histograms in Figure 5, which show how often a certain number of molecules were dissolved in the opposite phase within the simulation time. The distributions depict very clear trends depending on the selected conditions. The left panels of Figure 5 show the histograms related to the number of MeOH molecules dissolved in DD. The top and bottom panels refer to the cases without and with LiCl, respectively, while colors refer to different concentration of TBP in the systems. To evaluate the influence of LiCl on the mixing, we compare the width of the distributions and the position of the maxima. In the presence of LiCl, the number of molecules in the opposite phase is decreased. This is especially clear when comparing the right tails of the distributions: in the LiCl‐free cases the tails extends to high numbers, up to 24 (not shown in the figure) at a TBP concentration of 60 molecules, whereas it stops at ten MeOH molecules in the presence of LiCl. Since in industrial extraction processes neat phase separation with low mutual miscibility of the solvents is mandatory to avoid solvent losses, according to these analyses the addition of LiCl might be a solution in cases in which the mutual miscibility of the solvents is too high.

The same evaluation was done for different TBP concentrations. The neat systems (red color) exhibit the lowest dissolution of MeOH molecules into DD, whereas the increased TBP concentration leads to enhanced migration of MeOH into DD. The maximum number of migrated MeOH molecules is found at the highest TBP concentration in the absence of LiCl. As mentioned above, mutual miscibility of the solvents should be avoided; therefore, according to the analysis, for this specific solvent pair the amount of TBP used for the extraction should be carefully evaluated to minimize solvent losses.

The right side of Figure 5 shows the same kind of analysis as the left but with respect to DD molecules migrated into MeOH. In general, the number of dissolved DD molecules is lower than the number of MeOH molecules dissolved in DD. The comparison of the top‐right and bottom‐right panels shows the strong effect of LiCl. Its presence hinders the migration of DD molecules into MeOH, in line with what we found in our previous work.^[19]^ The effect of TBP was less strong; nevertheless, we point out that, as expected, for higher concentrations of TBP the migration seems to be facilitated, as shown by the bars on the right side of both panels. Hence, with regard to the migration of DD into MeOH, LiCl might again be used to decrease mutual miscibility, whereas the amount of TBP should be carefully evaluated.

While the data depicted in Figure 5 illustrate distinct trends, it might be interesting to study the temporal evolution of the number of solvated molecules. These plots may be found in the Supporting Information.

To further analyze the migration of molecules into the other solvent, Table 2 lists the average numbers *n̄*_MeOH_ and *n̄*_DD_ of MeOH molecules solvated in DD and vice versa. In the case of MeOH an increase in *n̄*_MeOH_ can be observed on addition of TBP. Although no significant difference in migration is visible for systems with 15 or 30 TBP molecules, increasing the TBP concentration to 60 molecules leads to an average number of solvated molecules twice as high. Interestingly, the TBP concentration has no significant effect on DD migration. However, the addition of LiCl leads to a significant decrease in migration for both MeOH and DD, in accordance with our earlier observations.

**Table 2:** Average numbers *n̄*_MeOH_ and *n̄*_DD_ of MeOH molecules solvated in DD and vice versa, and average lifetimes $\tau_{\mathrm{MeOH}}\left(n\right)$ and $\tau_{\mathrm{DD}}\left(n\right)$ [ps] of states in which at least *n* (or 0) molecules of MeOH or DD are solvated in the other solvent, measured in the CUB systems.

System	*n̄*_MeOH_	*n̄*_DD_	$\tau_{\mathrm{MeOH}}\left(0\right)$	$\tau_{\mathrm{MeOH}}\left(1\right)$	$\tau_{\mathrm{MeOH}}\left(2\right)$	$\tau_{\mathrm{DD}}\left(0\right)$	$\tau_{\mathrm{DD}}\left(1\right)$	$\tau_{\mathrm{DD}}\left(2\right)$
MD	2.1	1.5	7.0	17.2	9.1	9.4	34.4	13.1
MDT15	2.5	1.4	8.5	44.9	16.3	5.8	20.4	8.6
MDT30	2.8	1.4	2.9	24.7	9.6	4.7	18.2	9.1
MDT60	5.7	1.3	0.5	227.5	63.9	7.0	17.8	9.1
MLD	1.1	0.0	9.7	18.1	10.0	115.9	5.8	–
MLDT15	1.5	0.0	9.8	19.5	13.8	2841.8	0.8	–
MLDT30	1.4	0.1	7.1	16.1	9.7	105.3	6.6	–
MLDT60	3.0	0.3	2.6	71.0	18.5	20.0	9.5	3.5

Average lifetimes $\tau_{\mathrm{MeOH}}\left(n\right)$ and $\tau_{\mathrm{DD}}\left(n\right)$ of states in which at least *n* (or 0) molecules of MeOH or DD are solvated in the other solvent are listed in Table 2. The same trends can be observed, whereby a high TBP concentration increases the lifetime of solvated molecules and the addition of LiCl drastically reduces it.

We note that, since the domain analysis included TBP as Voronoi sites, it is possible that molecules solvated by TBP rather than the opposite solvent are counted toward the number of migrated molecules. However, if TBP is excluded, molecules solvated by the opposite solvent and separated by TBP might be counted toward the main domain. It is clear that the true number of migrated molecules must lie in between. Hence, we carried out additional analyses that excluded TBP as Voronoi sites, which can be found in the Supporting Information. Although the number of migrated molecules decreases, similar trends are observed, that is, the observed effects on mutual miscibility are not merely due to solvation by TBP.

**Figure 6:**
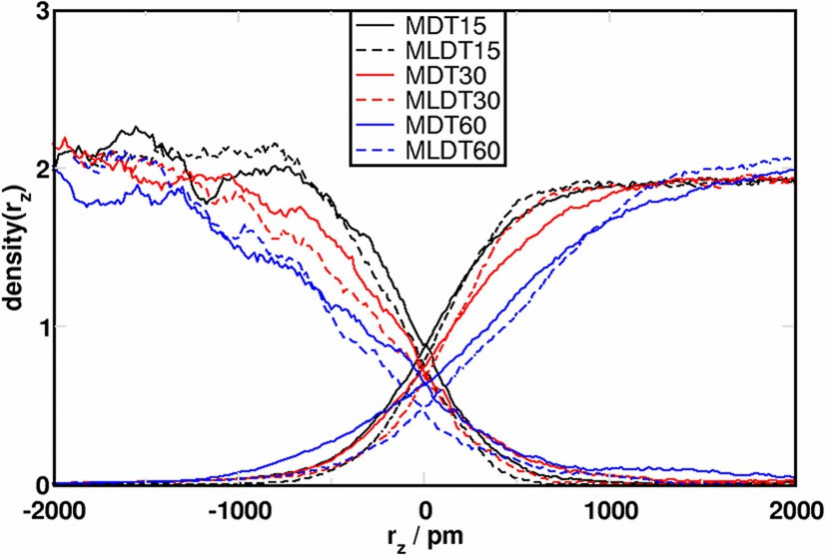
Normalized density profiles of the solvents in NCUB systems. Density profile curves starting high on the left refer to DD, and those on the right to MeOH.

Next, we turn to the systems that were set up with an interface (see Figure 3). Figure 6 shows the normalized density profiles of the solvents, centered at the interface calculated for NCUB systems. An ideal biphasic system would show a neat and sudden drop of the density of the solvents at the interface position. Changes in the concentration of TBP, indicated by different colors in the plots, strongly affect the behavior of the solvents at the interface. We focus first on the curves describing MeOH density (the curves starting at about 2 on the right), as the noise in the DD curves makes their evaluation complicated. For higher concentrations of TBP the density curves of each solvent on the solvent phase side were lower, whereas they were higher on the other side. As observed in the previous analyses, this effect was counteracted by the addition of LiCl, as it decreases the densities of the solvents in the opposite phase and increases the densities of the solvents in their own phase. This means that TBP increases mutual miscibility in the vicinity of the interface, whereas LiCl can be used to reduce mutual miscibility. This interfacial exchange is in line with the mixing behavior observed in the Voronoi analyses and it provides a good alternative tool to study solvent miscibility from a different and complementary perspective, as it provides information on the behavior of the solvents at the interface.

### Interface structure

In the previous section it was determined how TBP concentration affects the mutual miscibility of the solvents by increasing the exchange of molecules between the two phases. Therefore, the positioning of TBP molecules was studied by visual analysis of the trajectories of the CUB systems. In Figures 1 and 2 two snapshots of the systems after equilibration are depicted, which represent the general situations over the whole simulation time. Distinctively biphasic systems were obtained. From visual inspection it is apparent that in both cases the TBP molecules are adsorbed at the interface. Therefore, a further investigation of TBP adsorption at the interface seems of paramount importance. With this aim, we turn again to the NCUB systems. Figure 3 shows the simulation box of system NCUB‐MDT30. As expected TBP was mainly adsorbed at the interface between the solvents, and phase separation was maintained during the whole simulation time. From the snapshot we also obtained a first glimpse of the possibility of desorption of TBP into each solvent.

**Figure 7:**
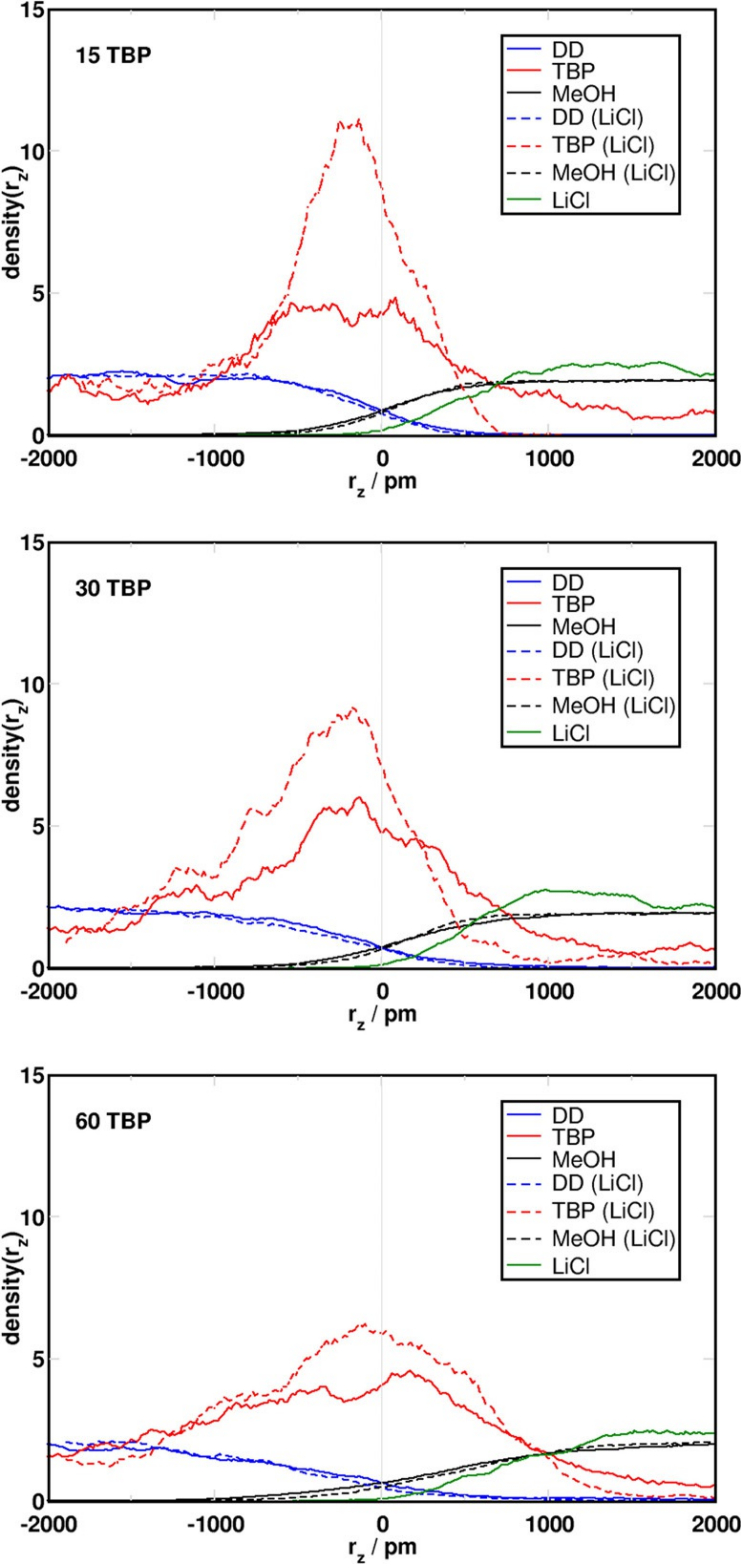
Normalized density profiles of the solvents and TBP in NCUB systems. Black: MeOH, blue: DD, green: LiCl and red: TBP. Upper panel:15 TBP, middle panel: 30 TBP, and bottom panel: 60 TBP.

Figure 7 shows the density profiles of solvents and TBP along the *z* direction. The solid red curves represent TBP density along the *z* direction in systems without LiCl, and the dashed red lines the TBP density in presence of the salt. The green solid line represents the density along the *z* direction of LiCl, and black and blue lines describe the density along the *z* direction of MeOH and DD (i.e., the MeOH phase starts from 2000 pm, reaching into the DD phase, and vice versa), respectively. The three panels report information for different concentrations of TBP, which was increased from top to bottom. TBP exhibits very different behaviors depending on its concentration and the presence of LiCl. For increasing concentrations of TBP, which was shown before to result in a negative effect on phase separation, we find broader density profiles, suggesting the mixing of TBP into MeOH and DD, at least in the vicinity of the interface. LiCl, on the other hand, tends to increase the density of TBP in the interfacial region. This can be explained by analyzing the shape of the density curves in the surrounding of the interface. We observe that LiCl decreases the density of TBP on the MeOH side, whereas it increases on the interface and remains the same on the DD side. Hence, this effect could be related to a hindrance of the diffusion of TBP molecules into MeOH due to LiCl, the subsequent accumulation of TBP at the interface, and, when the interface is saturated, into DD.

**Figure 8:**
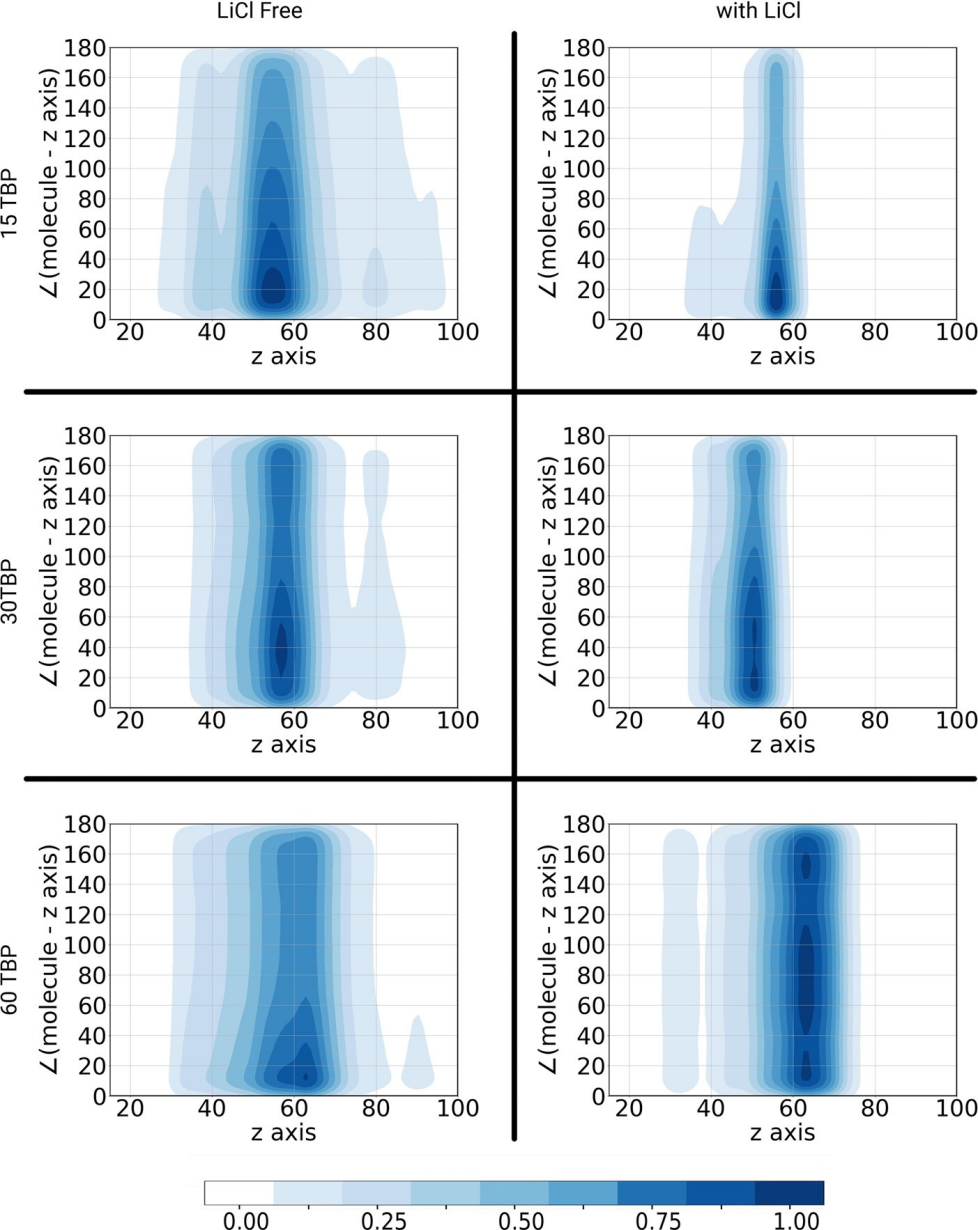
Combined distribution functions of TBP position and orientation in NCUB systems. The *x* direction of each graph represents the distribution of TBP molecules along the *z* axis of the simulation (×10^2^ pm) defined by the position of the P atom; the *y* axis is the angle α formed between the *z* axis of the simulation and the P=O bond of TBP. Therefore, values close to 180 ° represent the orientation of the polar moiety toward the DD phase, and 0 ° toward the MeOH phase. The DD phase is depicted on the left side of the graphs, and MeOH phase on the right side.

To further study the structural features of the interface, we show in Figure 8 the correlation between the positioning and the orientation of TBP molecules. The *x* axis represents the position of TBP molecules during the simulation, along the *z* direction defined by the position of the P atom, while the orientation, which is defined as the angle α between the *z* axis of the simulation and the P=O bond of TBP (see Figure 4) is reported on the *y* axis. Further visualizations of this analysis including cosα and the order parameter $S=\frac{3{cos}^{2}\alpha -1}{2}$ can be found in the Supporting Information. Additionally, similar analyses of the MeOH orientation in dependence on its position along the *z* axis were carried out and can be found in the Supporting Information with a set of combined distribution functions of the hydrogen‐bond angle and distance between MeOH and TBP. For values of α close to 180°, the orientation of the molecule has the polar moiety pointing toward the DD phase, and for values close to 0° an orientation toward the MeOH phase is indicated. As expected, TBP is found preferentially at the interfacial region between the two liquids (at approximately 60 000 pm), which confirms the above observation that it is adsorbed at the interface. Figure 8 also allows the comparison of interfacial TBP orientation depending on its concentration and the presence of LiCl. In the case without LiCl, TBP occupies a wider portion of space, and diffusion into both phases seems possible. When TBP is adsorbed at the interface, it exhibits a preferential orientation with *α*≤40° in which the polar group points at the MeOH phase and the butyl groups at the DD phase. On the DD side of the interface, it is apparent that TBP lacks preferential orientation. On the MeOH side, however, orientations closer to 0° still seem to be slightly favored. As shown by the panels on the right side of Figure 8, the situation radically changes once LiCl is added. In fact, in the presence of LiCl, the TBP molecules are constrained to the interface on the MeOH side, whereas they still exhibit a certain degree of freedom on the DD side. Even more interesting is the effect of this constraint on the orientation of the molecules at the interface. We observe that, on increasing the number of TBP molecules, α is more evenly distributed between 0 and 180°, probably due to an excess of TBP molecules at the interface, which can be solved only by rearranging the interfacial structure itself. The most common orientation in the presence of 60 TBP molecules of *α*≈80° depicts a completely different situation, in which TBP is aligned parallel to the interface. The most reasonable interpretation of these analyses might be that, with the addition of LiCl to the polar phase, the excess of TBP molecules cannot diffuse into the MeOH phase. This is also evident in the reduced interaction between MeOH and TBP after addition of LiCl, which is visible in the combined distribution functions reported in the Supporting Information. Hence, TBP loses its preferential orientation towards the solvents, so that its P=O bond aligns with the *xy* plane and the interactions with other TBP molecules are maximized. This is important with regard to solvent extraction, because the lack of specific TBP orientation in systems containing LiCl most likely negatively affects a ligand–target extraction process. In fact, TBP would not expose the active side (the phosphate group) to the polar solvent, which is where the target is usually dissolved. On the other hand, for low concentrations of TBP the orientation seems to be maintained also in presence of LiCl, and therefore a balance between the concentration of LiCl and TBP might be the key to obtaining good phase separation without impeding the extraction process.

## Conclusion

We have reported molecular dynamics simulations of the interface between MeOH and DD in the presence of LiCl and TBP, which is a case study for solvent extraction in solvometallurgy. In the current work, we considered only one concentration of LiCl. Different salt concentrations might be the subject of further studies, since the concentration of LiCl must be carefully balanced against potentially detrimental effects such as reduced metal solubility in the polar phase or decreasing extraction efficiency by competition of Li^+^ with the extraction process of another metal ion. Additionally, salts other than LiCl may show similar effects and might be worthy of consideration. Indeed, our previous work suggests that the increased phase separation is not directly related to the nature of LiCl, but rather stems from a strengthening of the hydrogen‐bond network in the methanol phase.^[19]^ Thus, we expect similar results for other alkali metal halides. For industrial application of solvent extraction, one of the main requirements is neat phase separation between the solvents. Thus, with the cubic model of these systems and a newly developed analysis based on the Voronoi tessellation method, we evaluated the effect of TBP and LiCl on the mutual miscibility of the solvents. We found that higher TBP concentrations lead to enhanced mutual miscibility, and that LiCl counteracts this effect and improves phase separation. We observed that TBP is mainly adsorbed at the interface between the two solvents. Therefore, we focused on the interface and its features related to TBP and LiCl. With this aim, we built seven noncubic systems, starting with a flat and well‐fixed interface between the solvents in a specific position of the box. These simulations allowed us to study how the structural properties of the interface (related to adsorbed TBP molecules) vary depending on the chemical environment. By plotting the correlation between the position of TBP along the *z* axis and its orientation, we showed that TBP adsorption changed depending on its concentration and LiCl presence. Without LiCl, adsorbed TBP exhibits a well‐defined orientation, with its polar moiety oriented towards the MeOH phase and its alkyl chains pointing towards the DD phase, which maximizes the polar–polar interactions on one side and the nonpolar–nonpolar interactions on the other. For higher TBP concentrations this feature was preserved, and the TBP molecules that could not be adsorbed in the first layer at the interface owing to lack of space diffused into the solvents.

The presence of LiCl, which slows down the dynamics of the MeOH phase (as described in our previous work),^[19]^ hindered the diffusion of TBP molecules into the MeOH phase and increased the concentration of TBP at the interface. Also the orientation of interfacial TBP was affected, especially for higher concentrations of TBP. In fact, with LiCl, when the TBP concentration was increased, TBP migration into MeOH was hindered, and this constraint led to a rearrangement of the TBP layer, in which TBP molecules oriented towards each other to maximize the interaction of the polar and nonpolar moieties. All these effects can affect solvent extraction. In fact, in the absence of LiCl, the increased exchange of interfacial TBP molecules and a well‐defined orientation of TBP molecules most likely positively affect the formation of a ligand–target complex and its migration from one phase to the other. On the other hand, TBP was proven to increase the mutual miscibility of the solvents, which should be avoided in industrial application. Indeed, the works of Wipff et al. suggest that a saturated interface is necessary for extraction to occur, which limits the range in which TBP concentration can be optimized against detrimental effects such as solvent mixing. The addition of LiCl can improve the system by decreasing mutual miscibility, but it also interferes with the orientation of TBP, which might affect complex formation, and thereby hinder the extraction.

## Conflict of interest

The authors declare no conflict of interest. Open access funding enabled and organized by Projekt DEAL.

## Supporting information

As a service to our authors and readers, this journal provides supporting information supplied by the authors. Such materials are peer reviewed and may be re‐organized for online delivery, but are not copy‐edited or typeset. Technical support issues arising from supporting information (other than missing files) should be addressed to the authors.

Supplementary
