# Supplementary material for: Understanding the Complex Surface Interplay for Extraction: A Molecular Dynamics Study
Source: Chemistry. 2020 Oct 14;26(65):14969–77. doi: 10.1002/chem.202002744 (PMC7756757; doi:10.1002/chem.202002744)
Supplement: Supplementary file 1 — Supplementary [file CHEM-26-14969-s001.pdf]

# Chemistry–A European Journal

Supporting Information


## Understanding the Complex Surface Interplay for Extraction: A Molecular Dynamics Study


Roberto Macchieraldo, Johannes Ingenmey, and Barbara Kirchner*[a]

## Author Contributions

R.M. Methodology: Lead; Software: Lead; Visualization: Lead; Writing - Original Draft: Lead.

## S-1 Mutual dissolution of solvents

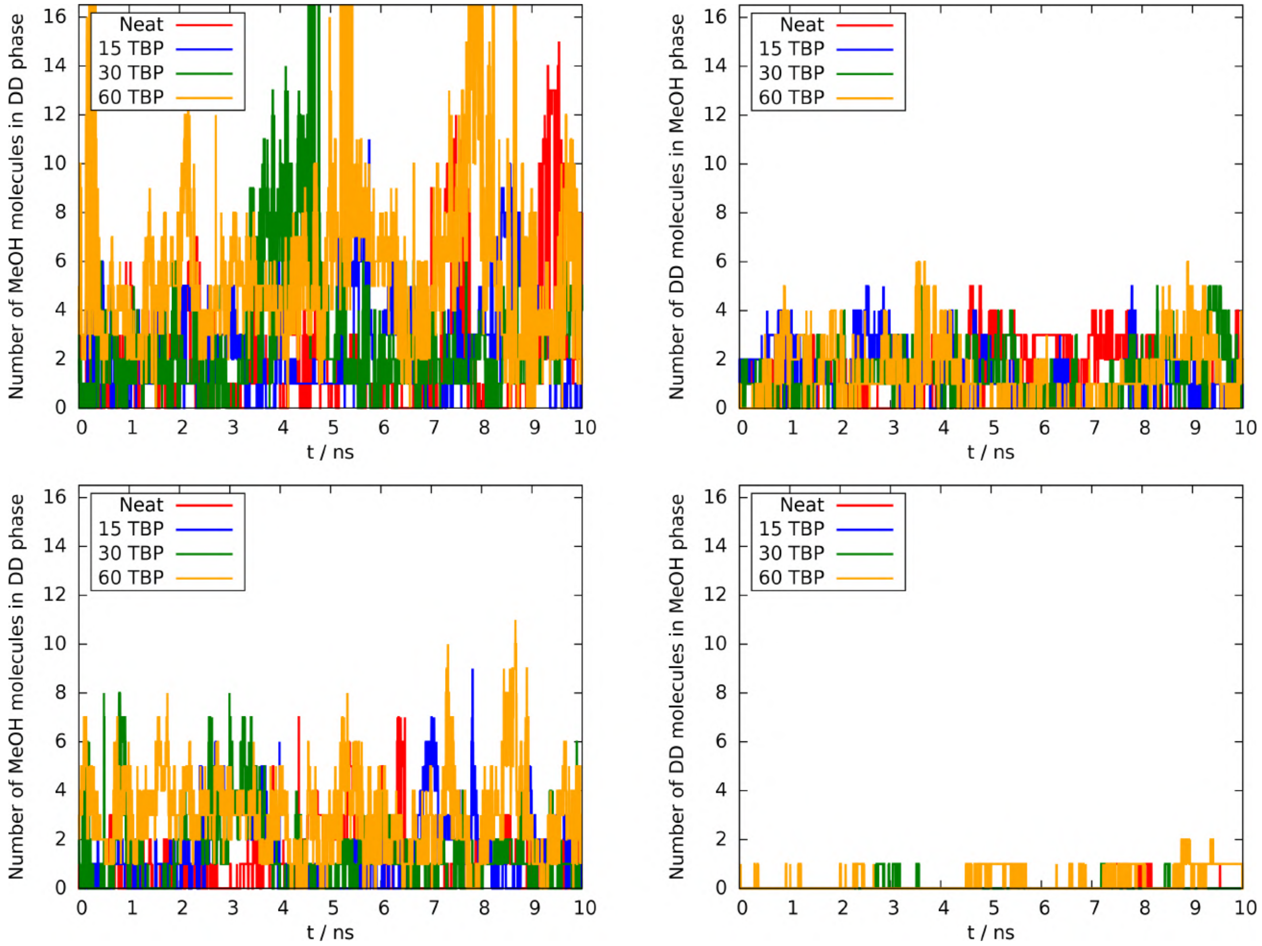


Figure S1: Temporal evolution of the number of molecules solvated by the other solvent. Left: Number of MeOH molecules migrated into the DD phase over the simulation time. Colors represent different TBP concentrations. Right: Number of DD molecules migrated into the MeOH phase over the simulation time. Top panels display the distribution in LiCl free system, bottom panels in systems with LiCl. Colors represent different TBP concentrations.

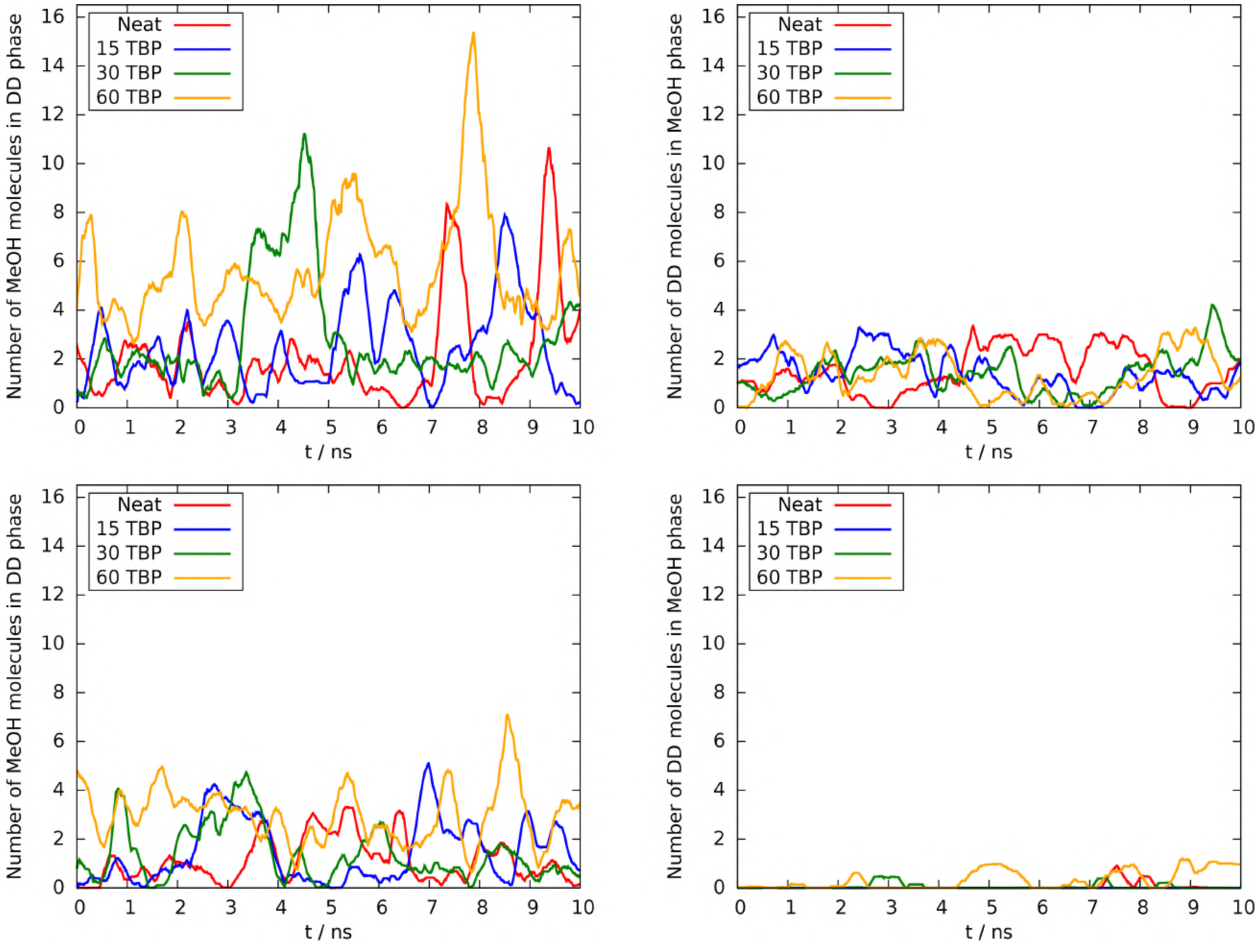


Figure S2: Moving average of the temporal evolution of the number of molecules solvated by the other solvent, taken over 300 time steps (0.15 ns). Left: Number of MeOH molecules migrated into the DD phase over the simulation time. Colors represent different TBP concentrations. Right: Number of DD molecules migrated into the MeOH phase over the simulation time. Top panels display the distribution in LiCl free system, bottom panels in systems with LiCl. Colors represent different TBP concentrations.

Table S1: Average lifetimes $\tau_{\mathrm{MeOH}}(n)$ (in ps) of states in which at least $n$ (or 0) molecules of MeOH are solvated in DD.

| n | MD | MDT15 | MDT30 | MDT60 | MLD | MLDT15 | MLDT30 | MLDT60 |
|---|---|---|---|---|---|---|---|---|
| 0 | 9.33 | 11.37 | 3.85 | 0.71 | 12.96 | 13.01 | 9.51 | 3.52 |
| 1 | 22.96 | 59.92 | 32.99 | 303.29 | 24.09 | 26.03 | 21.43 | 94.73 |
| 2 | 12.15 | 21.69 | 12.80 | 85.24 | 13.29 | 18.40 | 12.98 | 24.67 |
| 3 | 10.29 | 15.48 | 9.30 | 31.68 | 10.25 | 14.08 | 8.82 | 12.26 |
| 4 | 8.33 | 12.55 | 10.01 | 17.72 | 6.82 | 8.22 | 6.81 | 6.10 |
| 5 | 8.02 | 8.65 | 8.06 | 11.47 | 7.45 | 5.61 | 5.52 | 4.51 |
| 6 | 8.34 | 7.50 | 8.00 | 8.85 | 5.50 | 3.89 | 4.67 | 4.66 |

Table S2: Average lifetimes $\tau_{\mathrm{DD}}(n)$ (in ps) of states in which at least $n$ (or 0) molecules of DD are solvated in MeOH.

| n | MD | MDT15 | MDT30 | MDT60 | MLD | MLDT15 | MLDT30 | MLDT60 |
|---|---|---|---|---|---|---|---|---|
| 0 | 12.53 | 7.68 | 6.20 | 9.30 | 154.54 | 3789.00 | 140.41 | 26.68 |
| 1 | 45.88 | 27.20 | 24.31 | 23.77 | 7.74 | 1.00 | 8.85 | 12.72 |
| 2 | 17.49 | 11.51 | 12.07 | 12.13 | - | - | - | 4.63 |
| 3 | 11.51 | 7.05 | 7.30 | 5.80 | - | - | - | - |
| 4 | 5.43 | 3.43 | 7.07 | 4.09 | - | - | - | - |
| 5 | 3.67 | 4.14 | 4.00 | 3.27 | - | - | - | - |
| 6 | - | - | - | 1.73 | - | - | - | - |

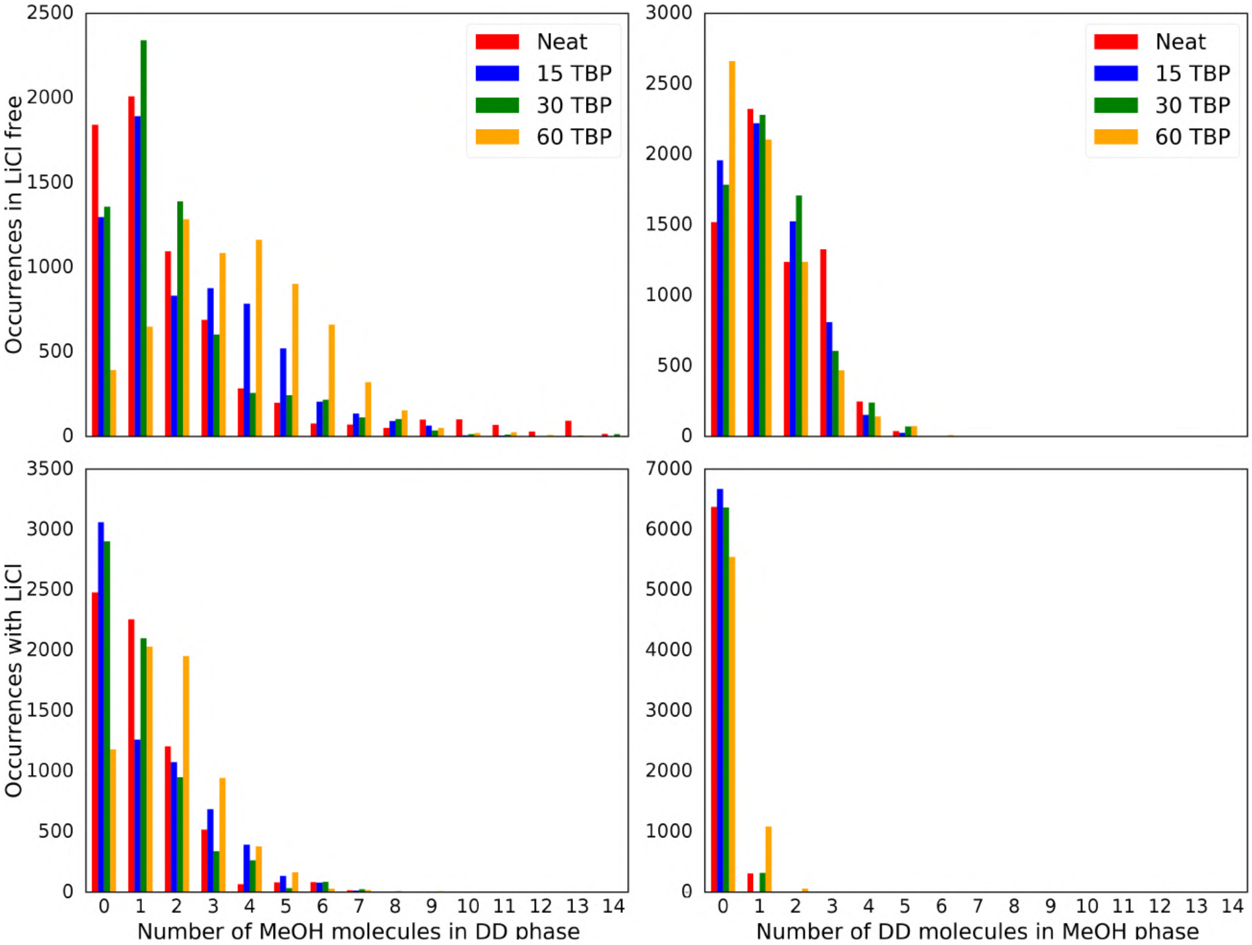


Figure S3: Distribution of molecules solvated by the other solvent as obtained *without* considering TBP as Voronoi site during the domain analysis. Left: Number of MeOH molecules migrated into the DD phase over the simulation time. Colors represent different TBP concentrations. Right: Number of DD molecules migrated into the MeOH phase over the simulation time. Top panels display the distribution in LiCl free system, bottom panels in systems with LiCl. Colors represent different TBP concentrations.

## S-2 Interface structure

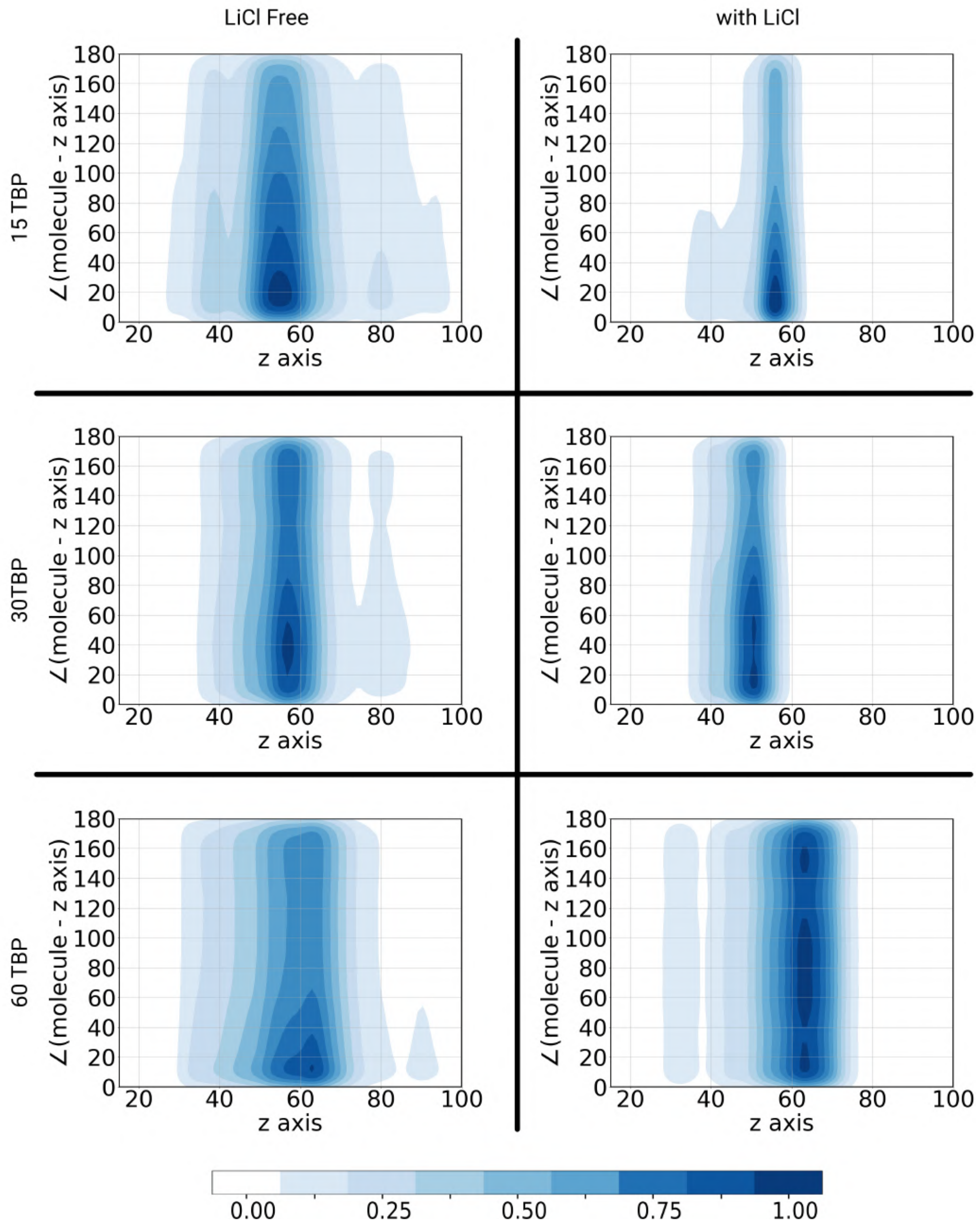


Figure S4: Combined distribution functions of TBP's position and orientation in NCUB-systems.The x direction of each graph represents the distribution of TBPs along the system's z axis ($*10^2$ pm) defined by the position of the P atom, the y axis is the angle $\alpha$ formed between the simulation z axis and the TBP's P=O bond. Therefore, values close to 180° represent the orientation of the polar moiety toward the DD phase, 0° toward the MeOH phase. The DD phase is depicted on the left side of the graphs, the MeOH on the right side.

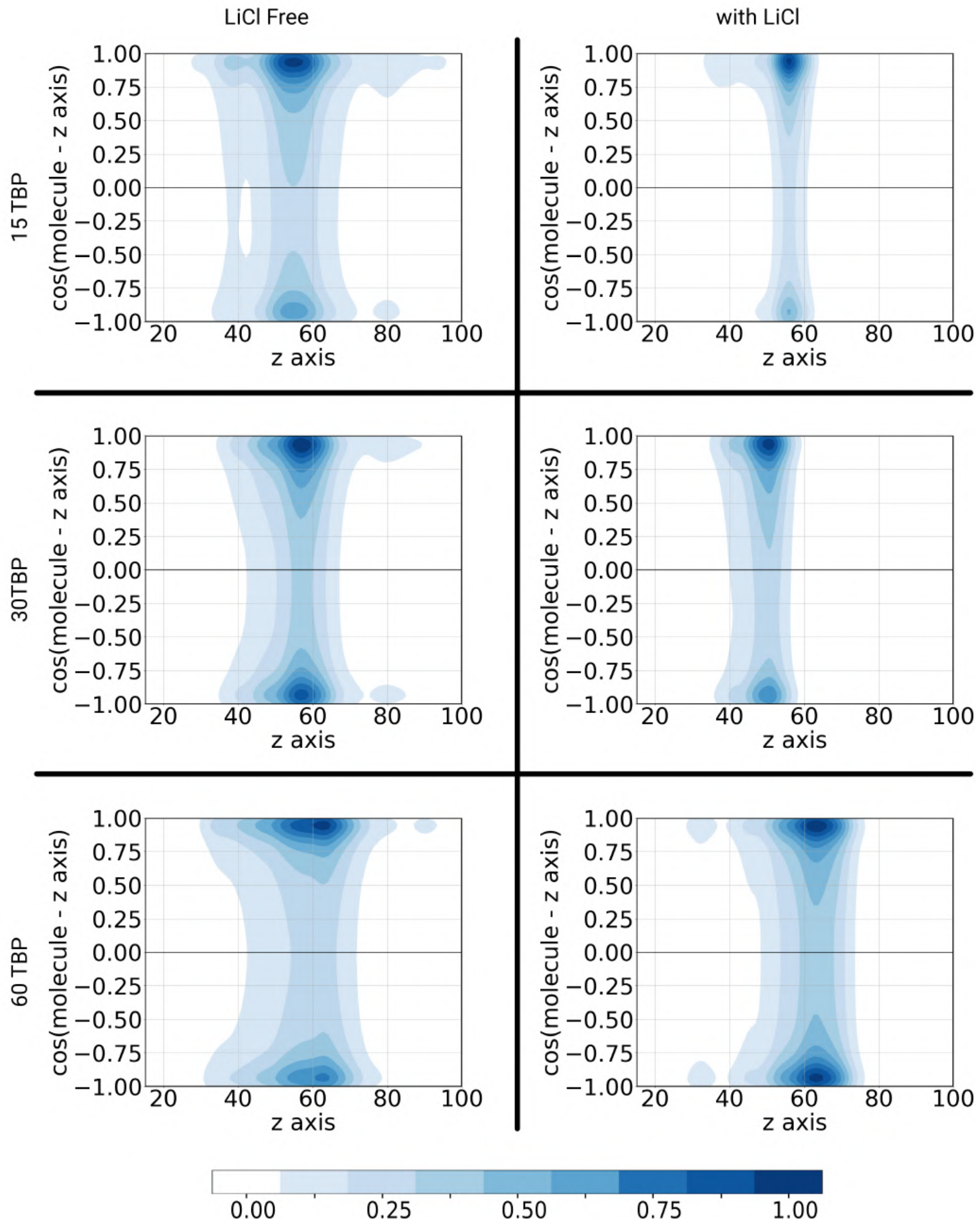


Figure S5: Combined distribution functions of TBP's position and orientation in NCUB-systems.The x direction of each graph represents the distribution of TBPs along the system's z axis ($*10^2$ pm) defined by the position of the P atom, the y axis is the cosine $\cos(\alpha)$ of the angle formed between the simulation z axis and the TBP's P=O bond. Therefore, values close to -1 represent the orientation of the polar moiety toward the DD phase, +1 toward the MeOH phase. The DD phase is depicted on the left side of the graphs, the MeOH on the right side.

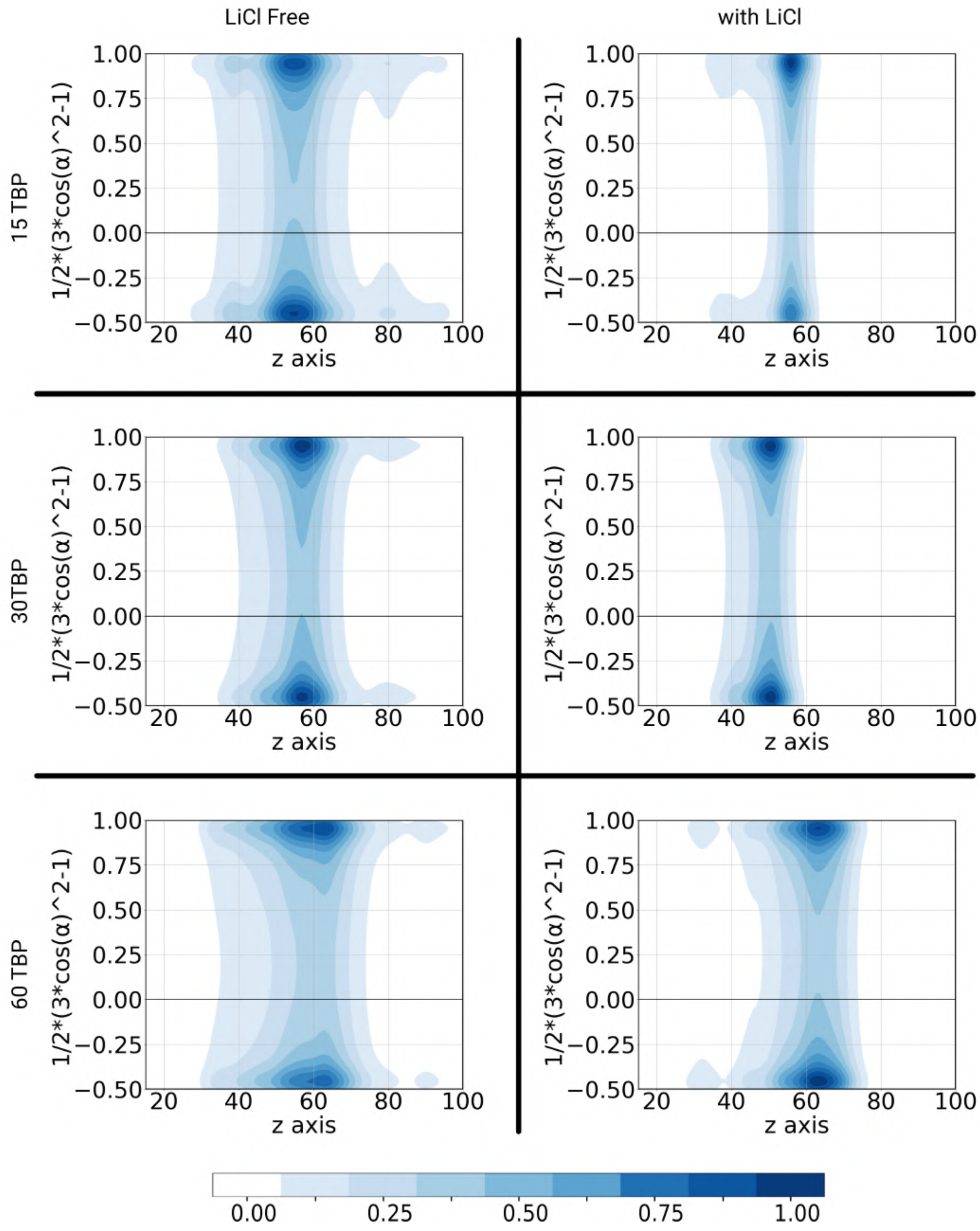


Figure S6: Combined distribution functions of TBP's position and orientation in NCUB-systems.The x direction of each graph represents the distribution of TBPs along the system's z axis ($*10^2$ pm) defined by the position of the P atom, the y axis is the orientational order parameter $S = \frac{3\cos^2\alpha - 1}{2}$ based on the angle $\alpha$ formed between the simulation z axis and the TBP's P=O bond. Therefore, values close to -0.5 represent an orientation perpendicular to the z axis and parallel to the interface, +1 parallel (or anti-parallel) to the z axis and perpendicular to the interface. The DD phase is depicted on the left side of the graphs, the MeOH on the right side.

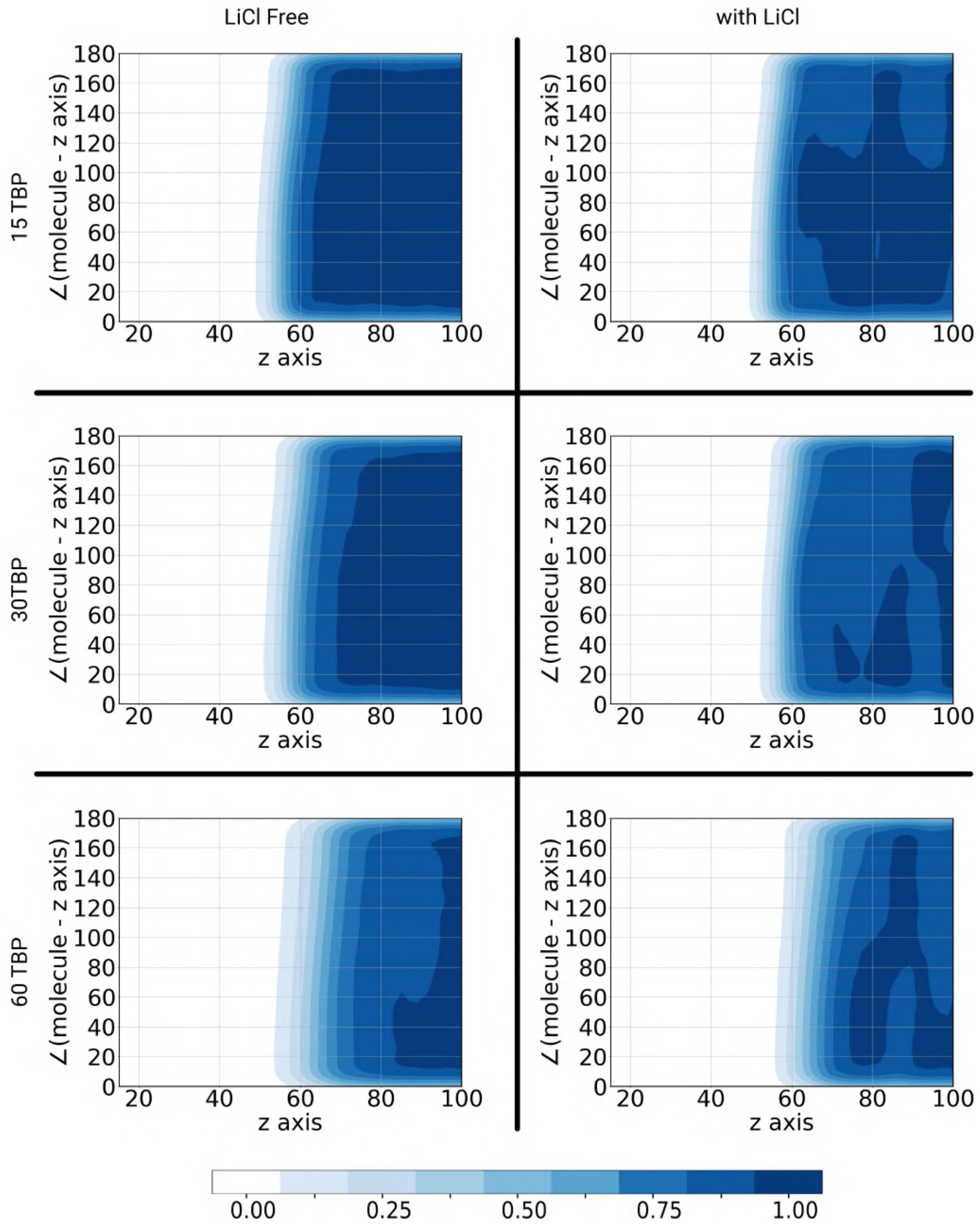


Figure S7: Combined distribution functions of MeOH's position and orientation in NCUB-systems.The x direction of each graph represents the distribution of MeOHs along the system's z axis ($*10^2$ pm) defined by the position of the O atom, the y axis is the angle $\alpha$ formed between the simulation z axis and the MeOH's O-H bond. Therefore, values close to 180° represent the orientation of the polar moiety toward the DD phase, 0° toward the MeOH phase. The DD phase is depicted on the left side of the graphs, the MeOH on the right side.

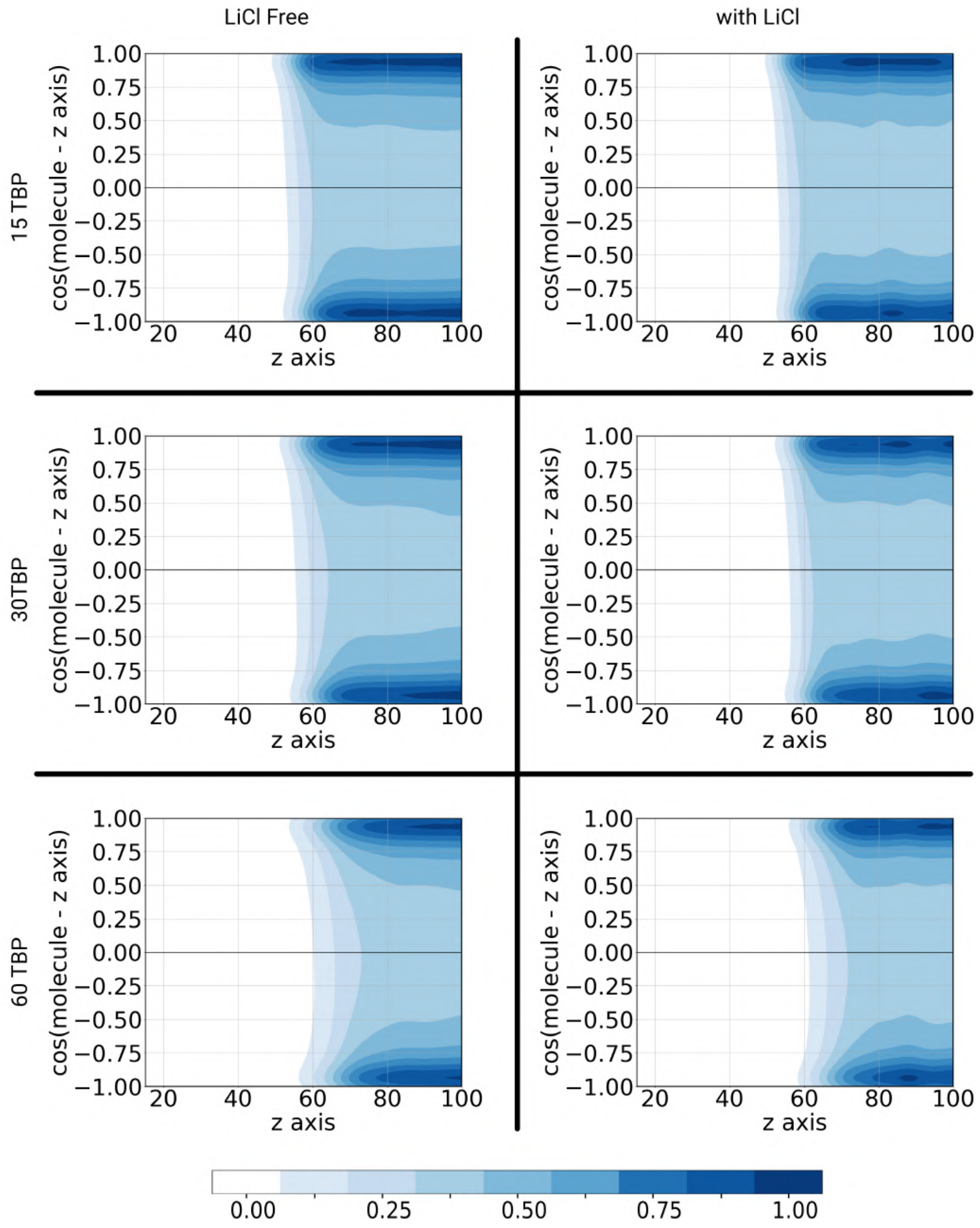


Figure S8: Combined distribution functions of MeOH's position and orientation in NCUB-systems.The x direction of each graph represents the distribution of MeOHs along the system's z axis ($*10^2$ pm) defined by the position of the O atom, the y axis is the cosine $\cos(\alpha)$ of the angle formed between the simulation z axis and the MeOH's O-H bond. Therefore, values close to -1 represent the orientation of the polar moiety toward the DD phase, +1 toward the MeOH phase. The DD phase is depicted on the left side of the graphs, the MeOH on the right side.

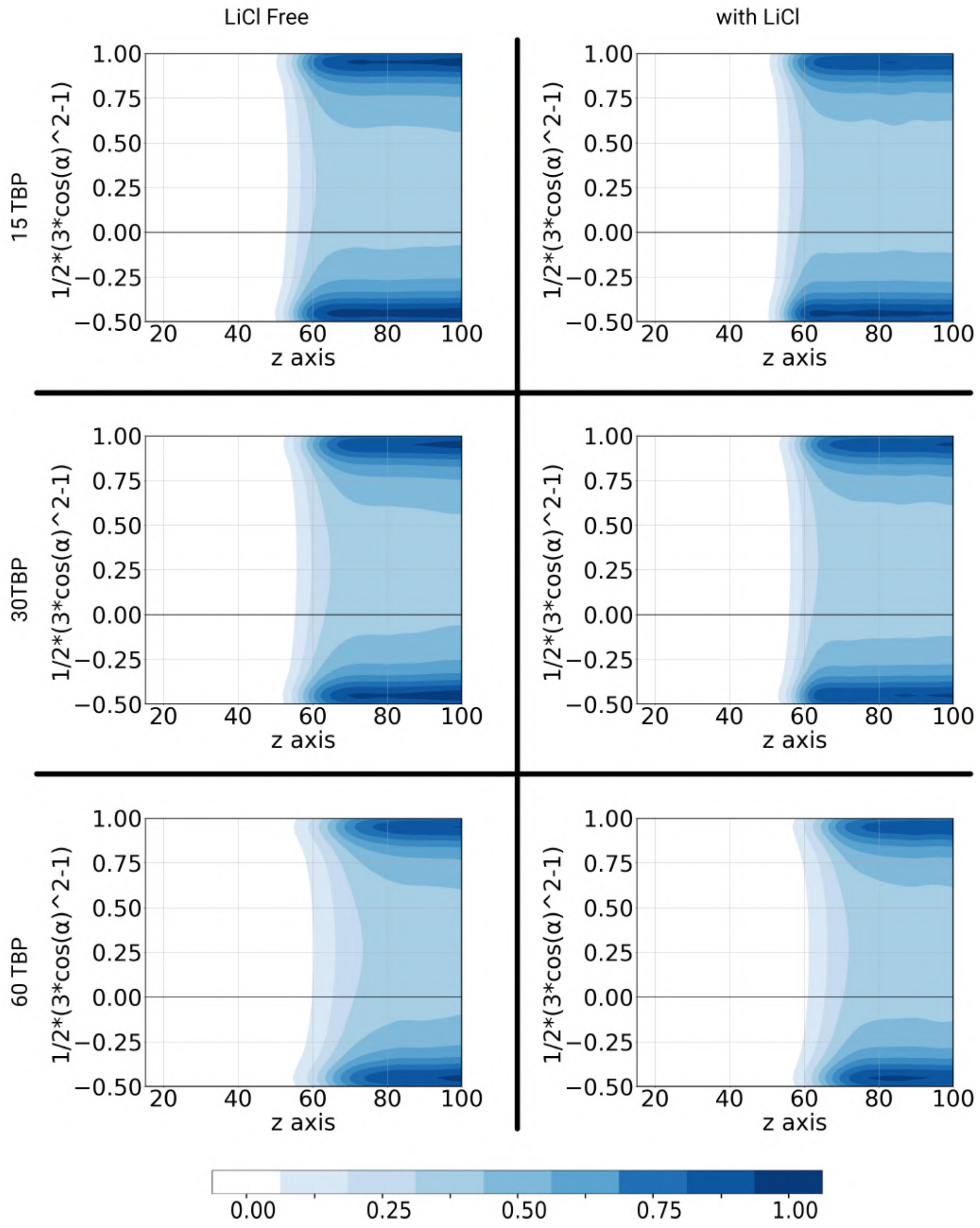


Figure S9: Combined distribution functions of MeOH's position and orientation in NCUB-systems.The x direction of each graph represents the distribution of MeOHs along the system's z axis ($*10^2$ pm) defined by the position of the O atom, the the y axis is the orientational order parameter $S = \frac{3\cos^2\alpha - 1}{2}$ based on the angle $\alpha$ formed between the simulation z axis and the MeOH's O-H bond. Therefore, values close to -0.5 represent an orientation perpendicular to the z axis and parallel to the interface, +1 parallel (or anti-parallel) to the z axis and perpendicular to the interface. The DD phase is depicted on the left side of the graphs, the MeOH on the right side.

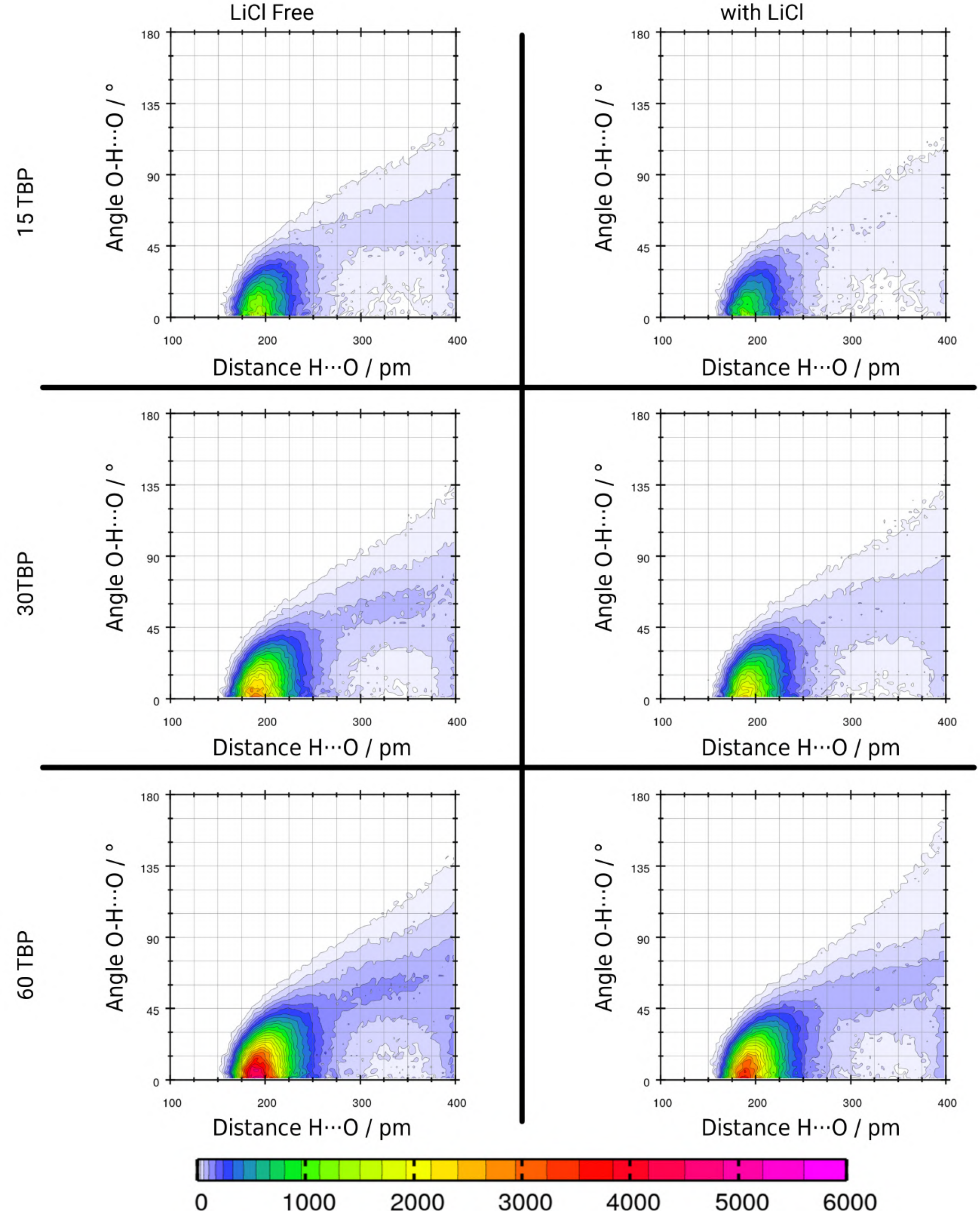


Figure S10: Combined distribution functions of the hydrogen bond angle and distance between MeOH and TBP. The occurrence in each CDF are scaled to each other so as to depict the effects of TBP and LiCl on the interaction between MeOH and TBP.

## S-3 Simulation Snapshots

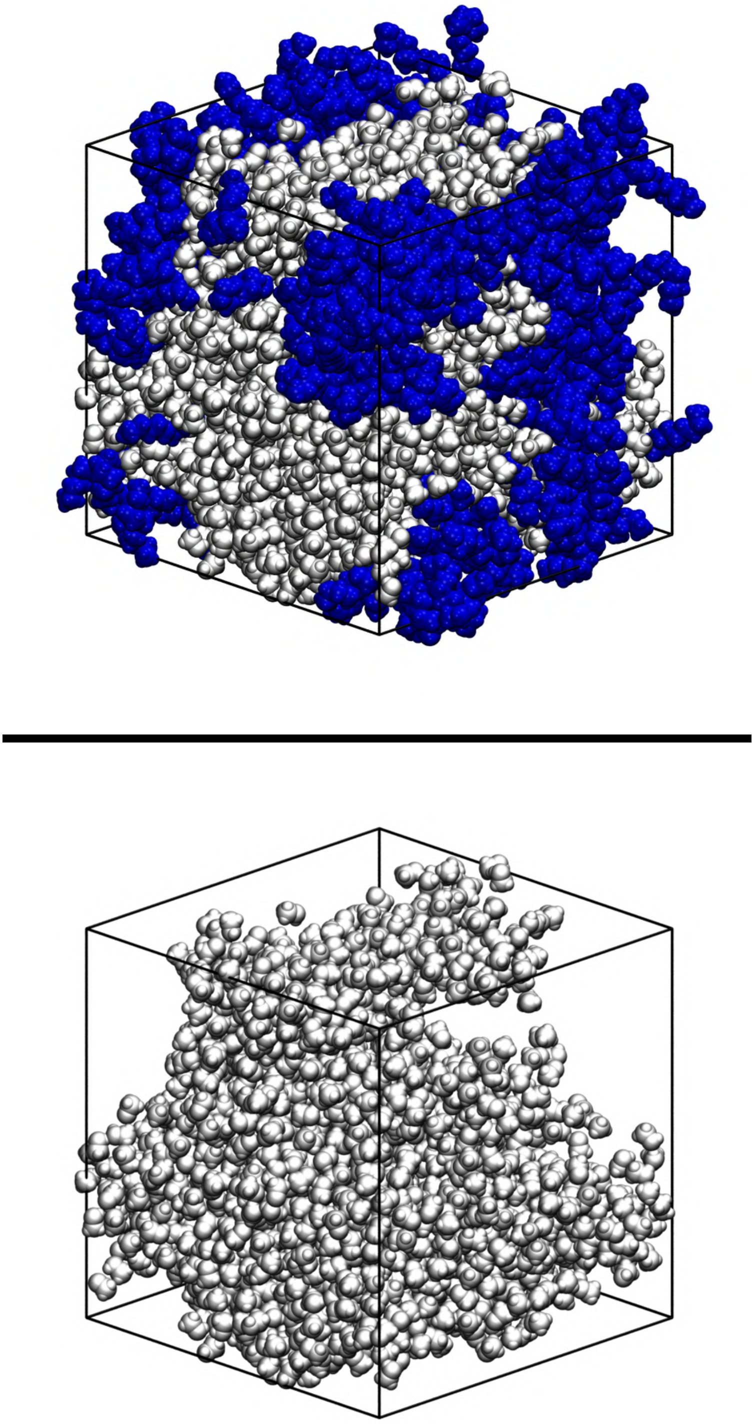

Figure S11: Snapshots of system CUB-MD. Atoms are represented by van der Waals spheres. White: MeOH, blue: DD. Top panel: full system, bottom panel: MeOH only.

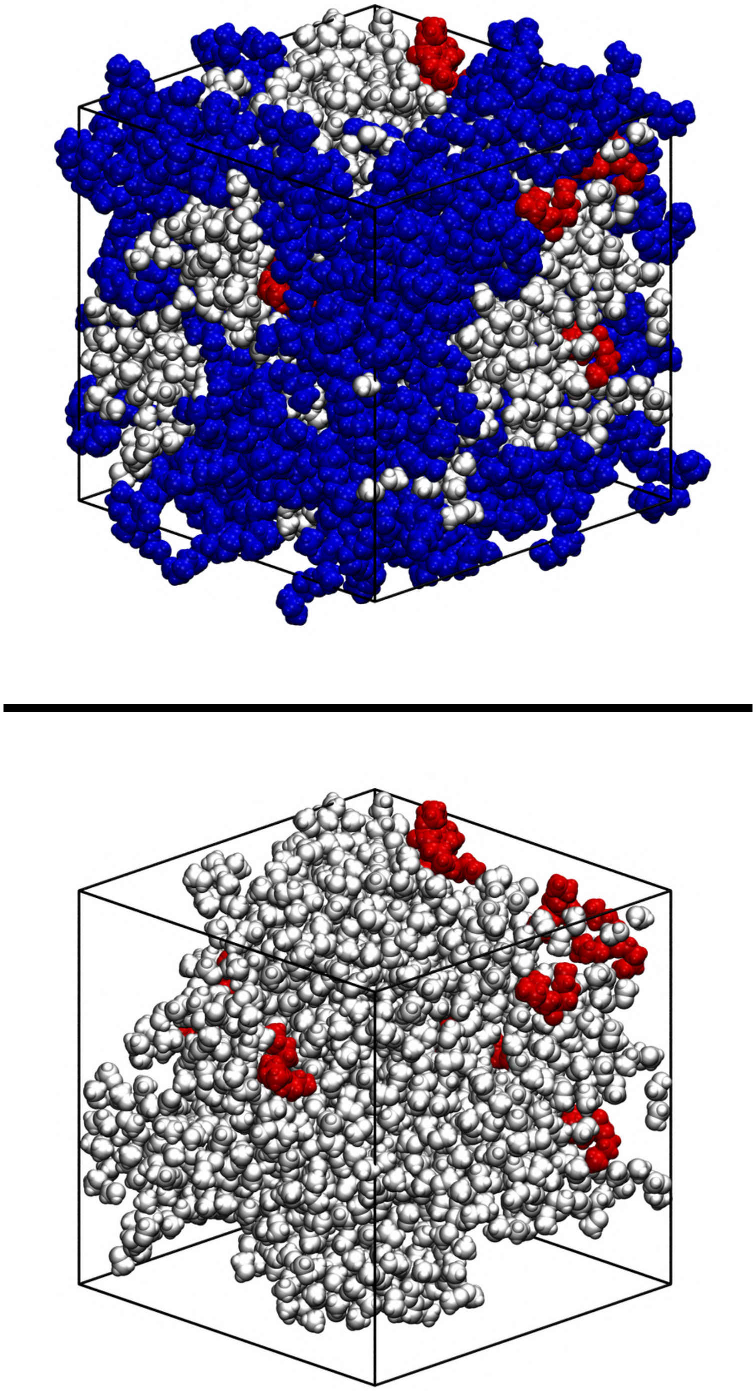

Figure S12: Snapshots of system CUB-MDT15. Atoms are represented by van der Waals spheres. White: MeOH, blue: DD, red: TBP. Top panel: full system, bottom panel: MeOH and TBP only.

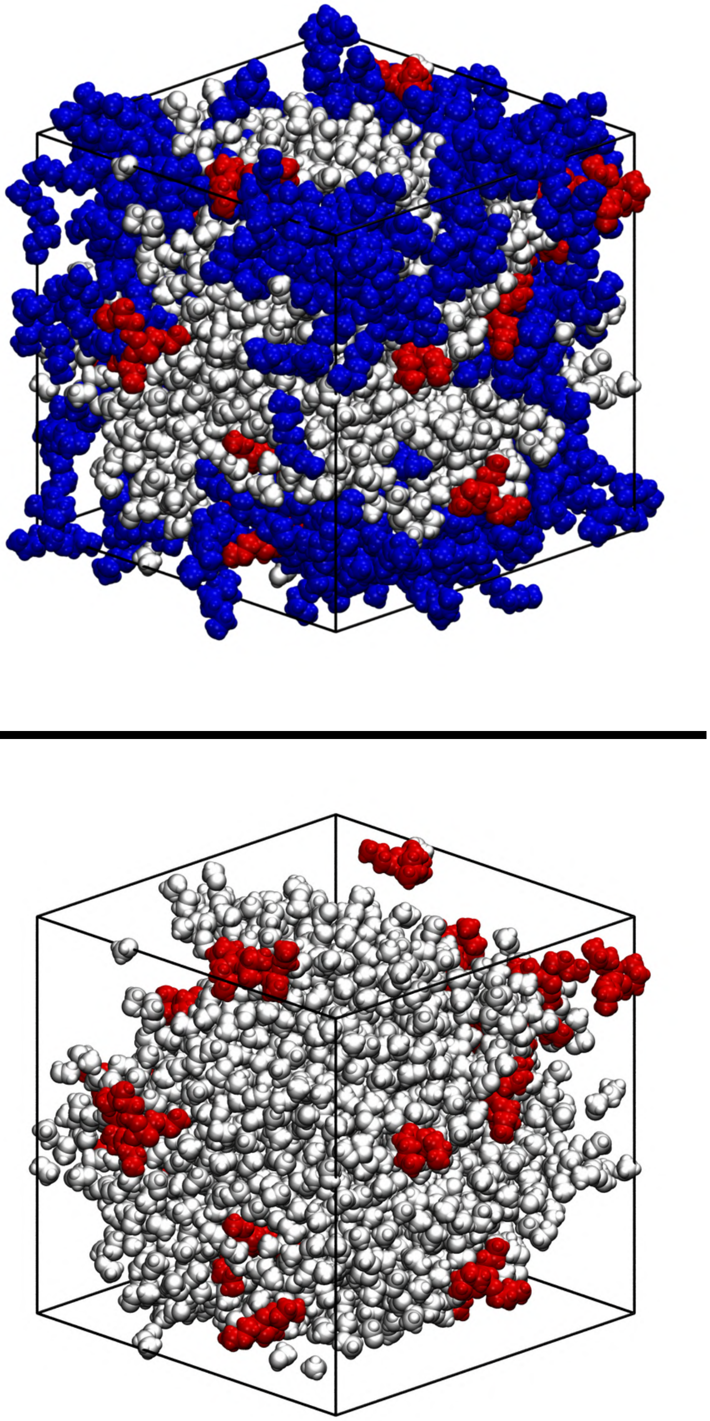

Figure S13: Snapshots of system CUB-MDT30. Atoms are represented by van der Waals spheres. White: MeOH, blue: DD, red: TBP. Top panel: full system, bottom panel: MeOH and TBP only.

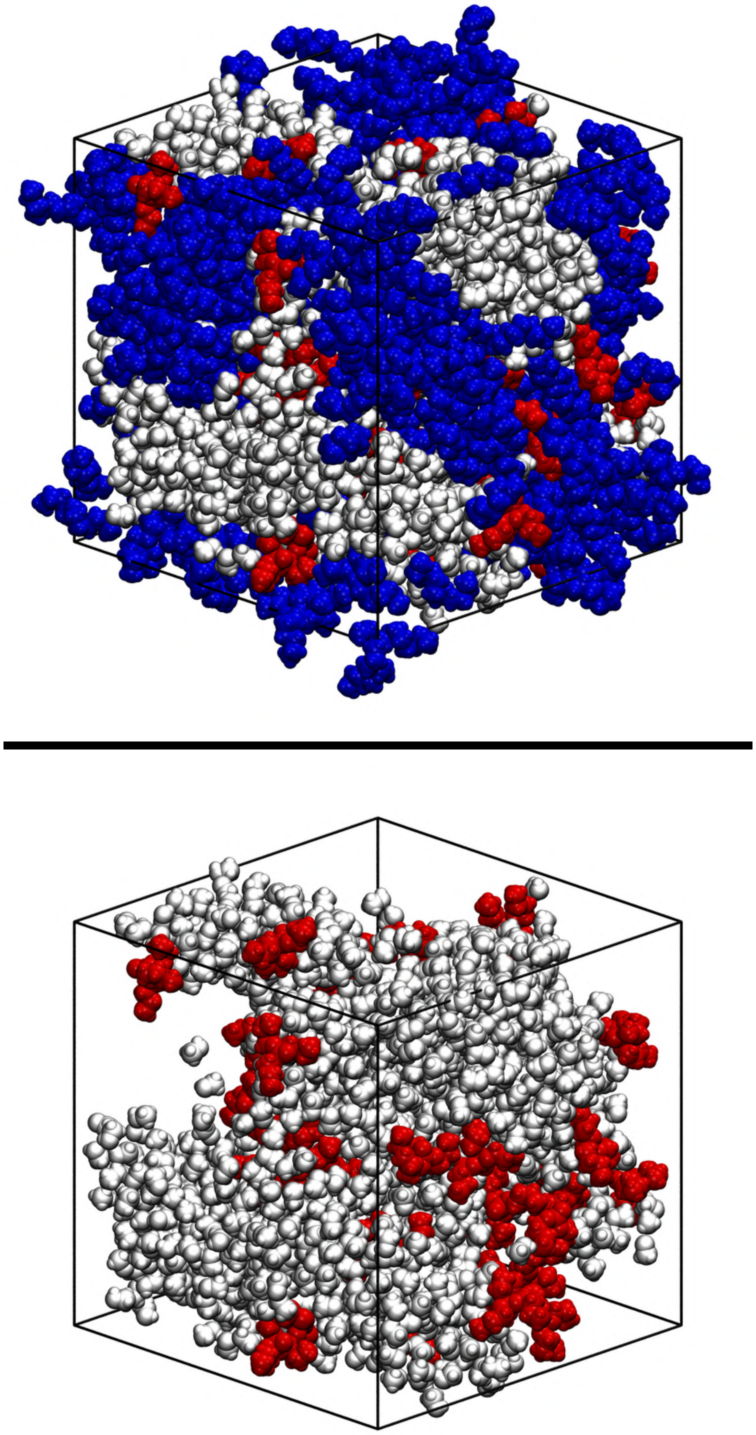

Figure S14: Snapshots of system CUB-MDT60. Atoms are represented by van der Waals spheres. White: MeOH, blue: DD, red: TBP. Top panel: full system, bottom panel: MeOH and TBP only.

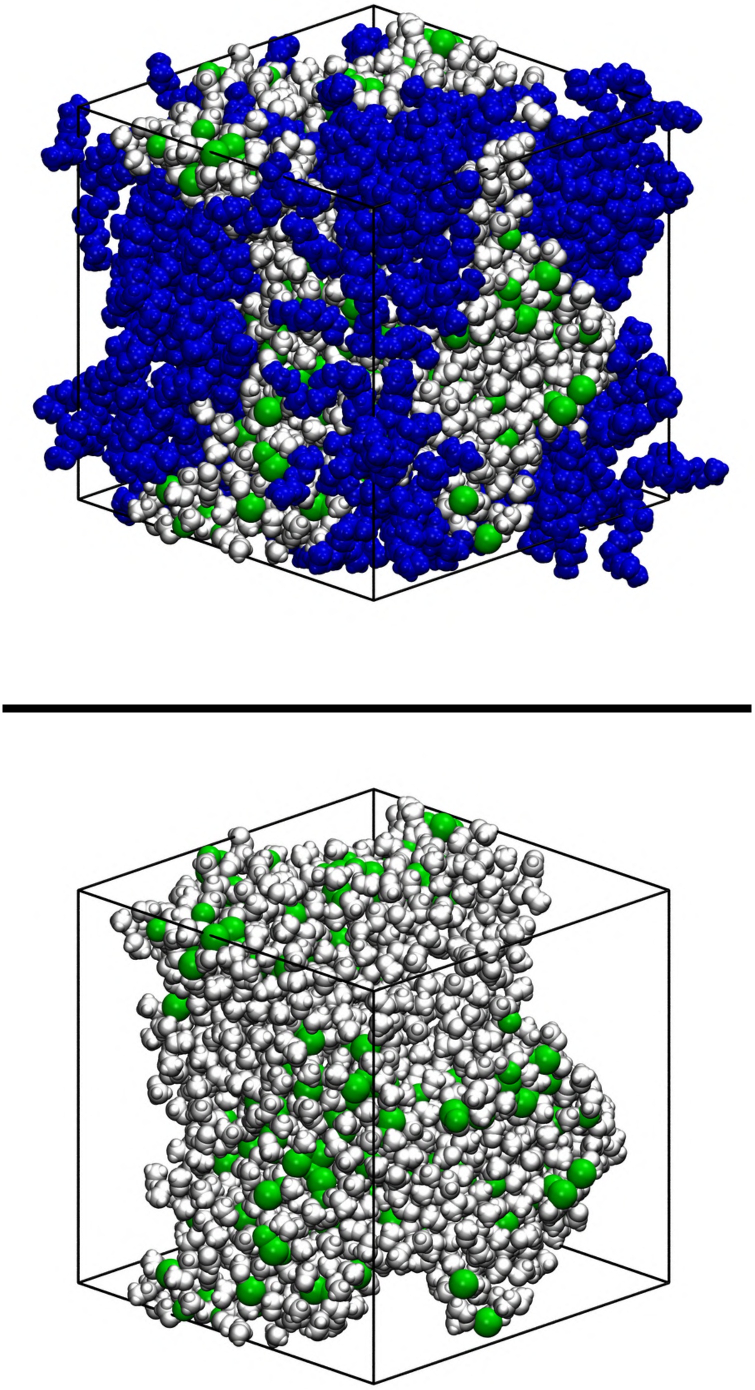

Figure S15: Snapshots of system CUB-MLD. Atoms are represented by van der Waals spheres. White: MeOH, blue: DD, green: LiCl. Top panel: full system, bottom panel: MeOH and LiCl only.

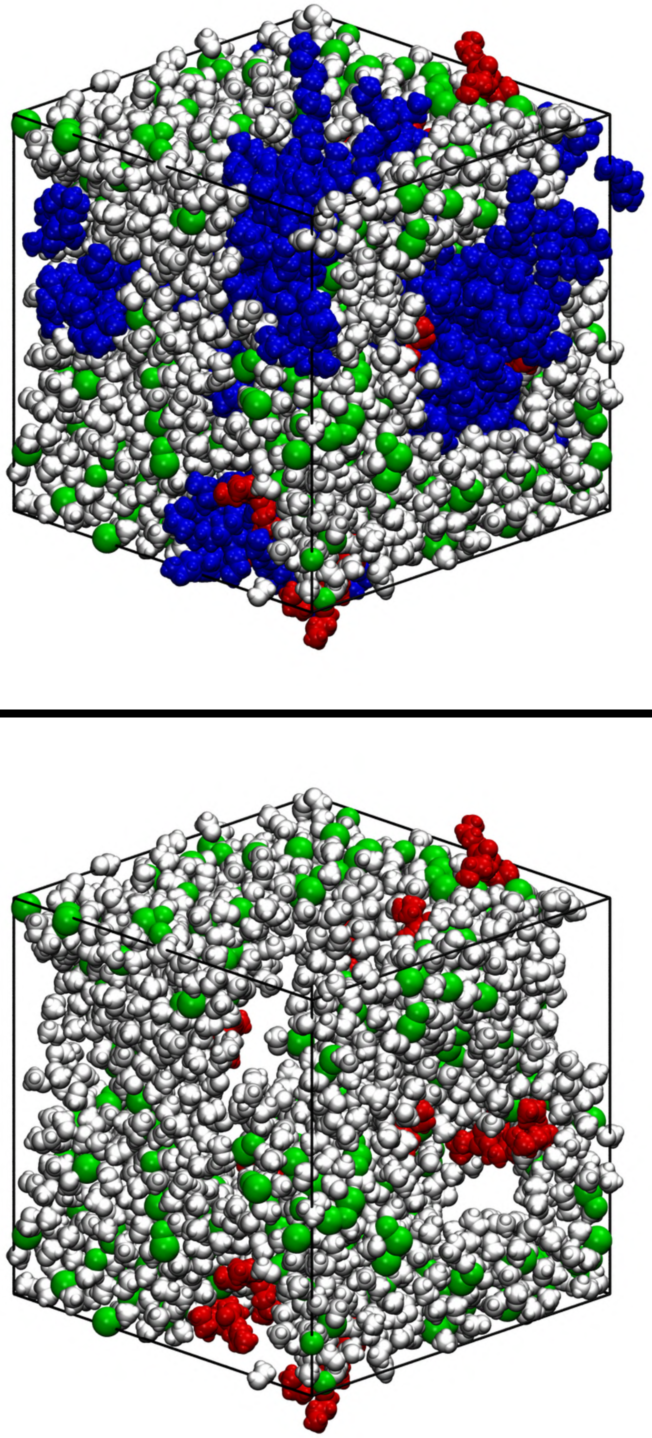

Figure S16: Snapshots of system CUB-MLDT15. Atoms are represented by van der Waals spheres. White: MeOH, blue: DD, red: TBP, green: LiCl. Top panel: full system, bottom panel: MeOH, LiCl and TBP only.

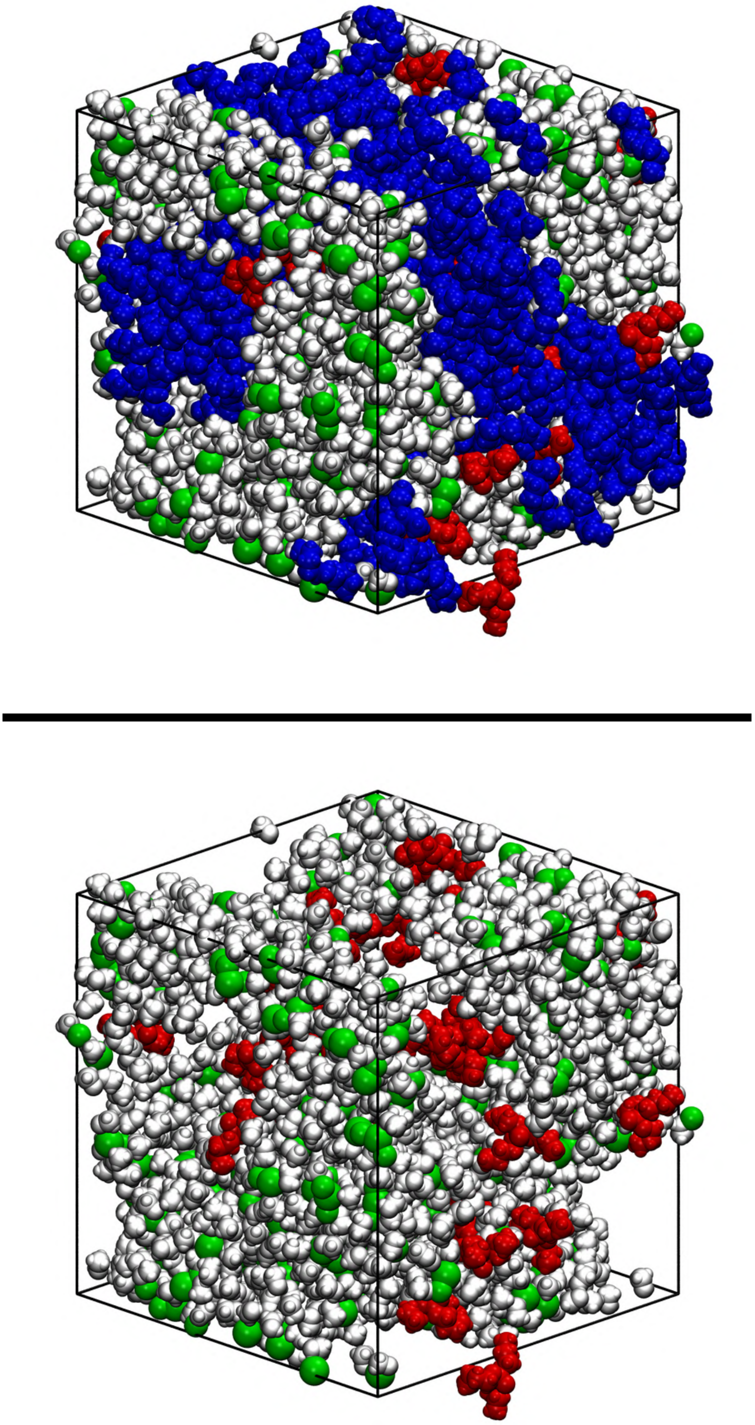

Figure S17: Snapshots of system CUB-MLDT30. Atoms are represented by van der Waals spheres. White: MeOH, blue: DD, red: TBP, green: LiCl. Top panel: full system, bottom panel: MeOH, LiCl and TBP only.

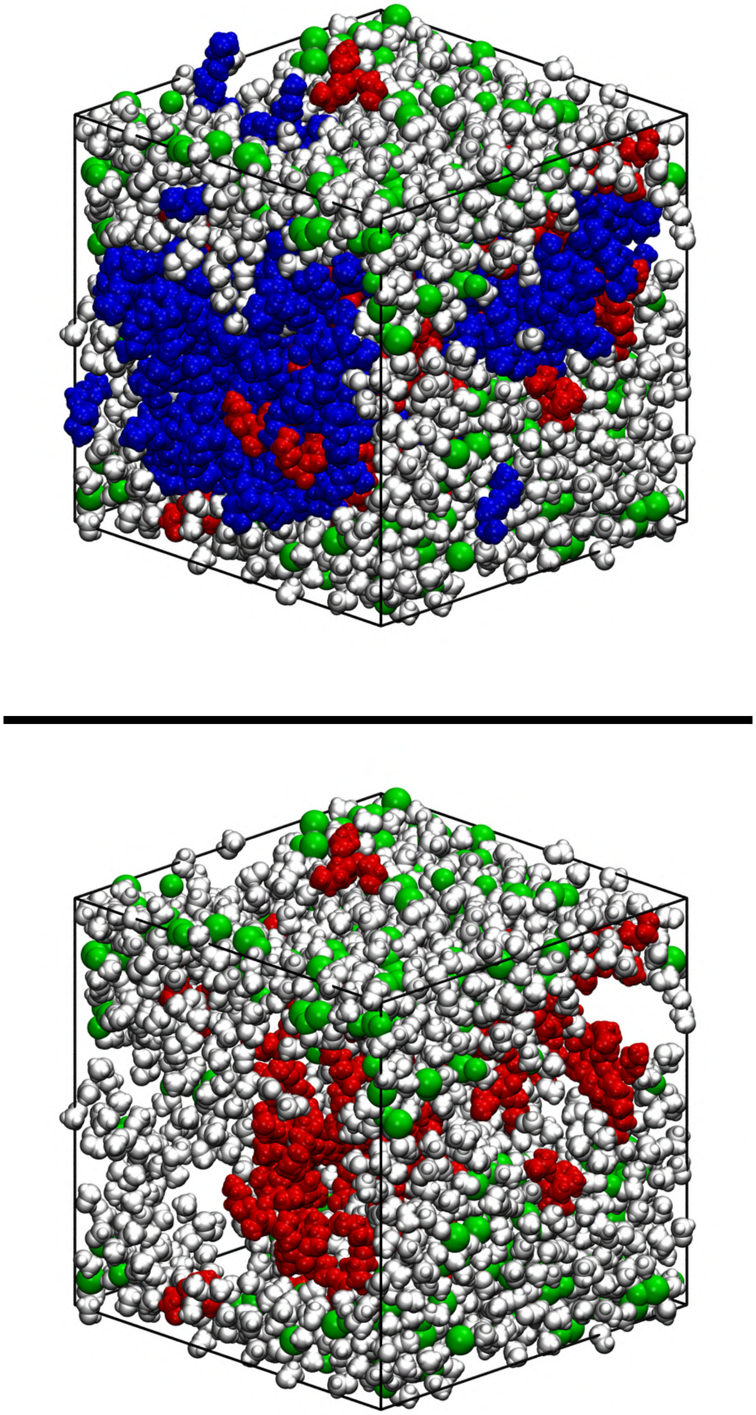

Figure S18: Snapshots of system CUB-MLDT60. Atoms are represented by van der Waals spheres. White: MeOH, blue: DD, red: TBP, green: LiCl. Top panel: full system, bottom panel: MeOH, LiCl and TBP only.

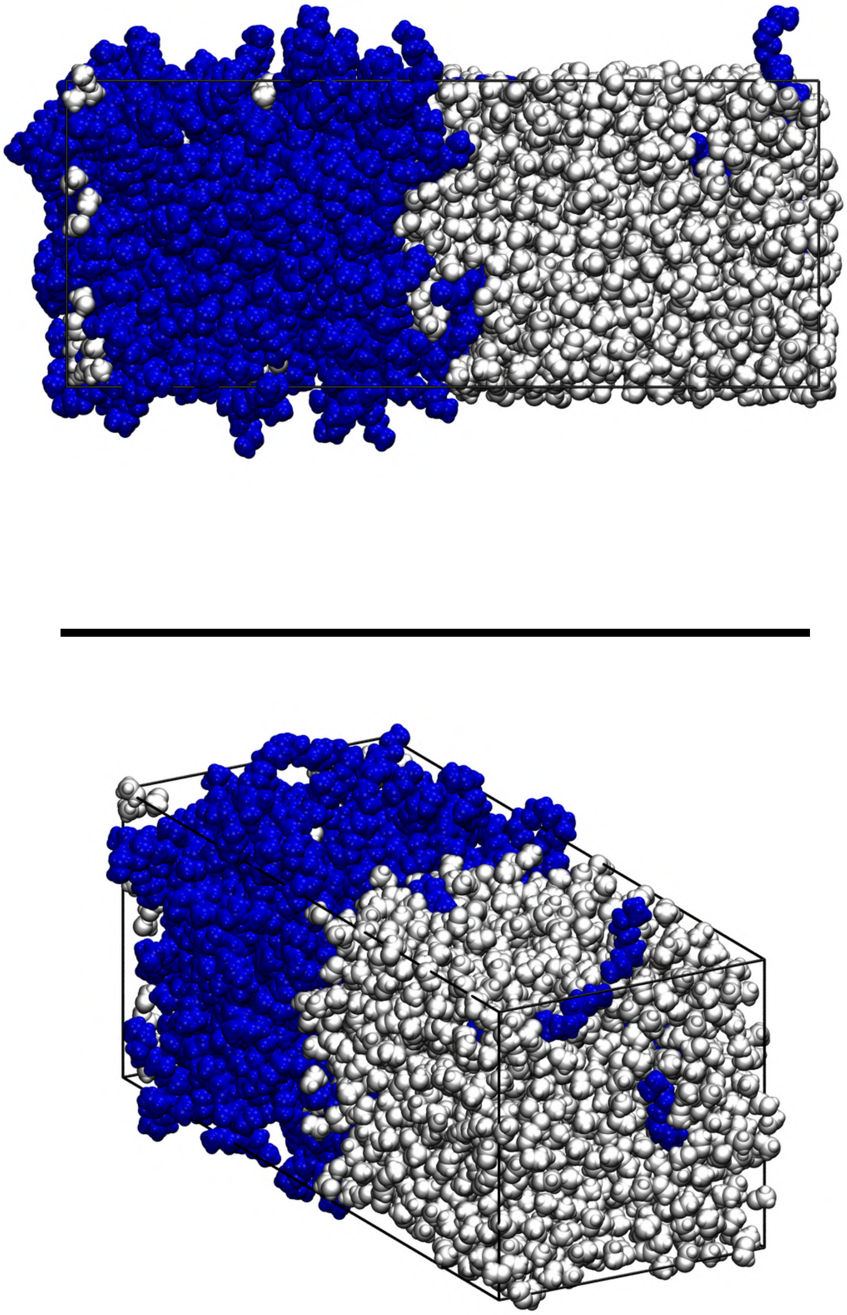

Figure S19: Snapshots of system CUB-MD. Atoms are represented by van der Waals spheres. White: MeOH, blue: DD.

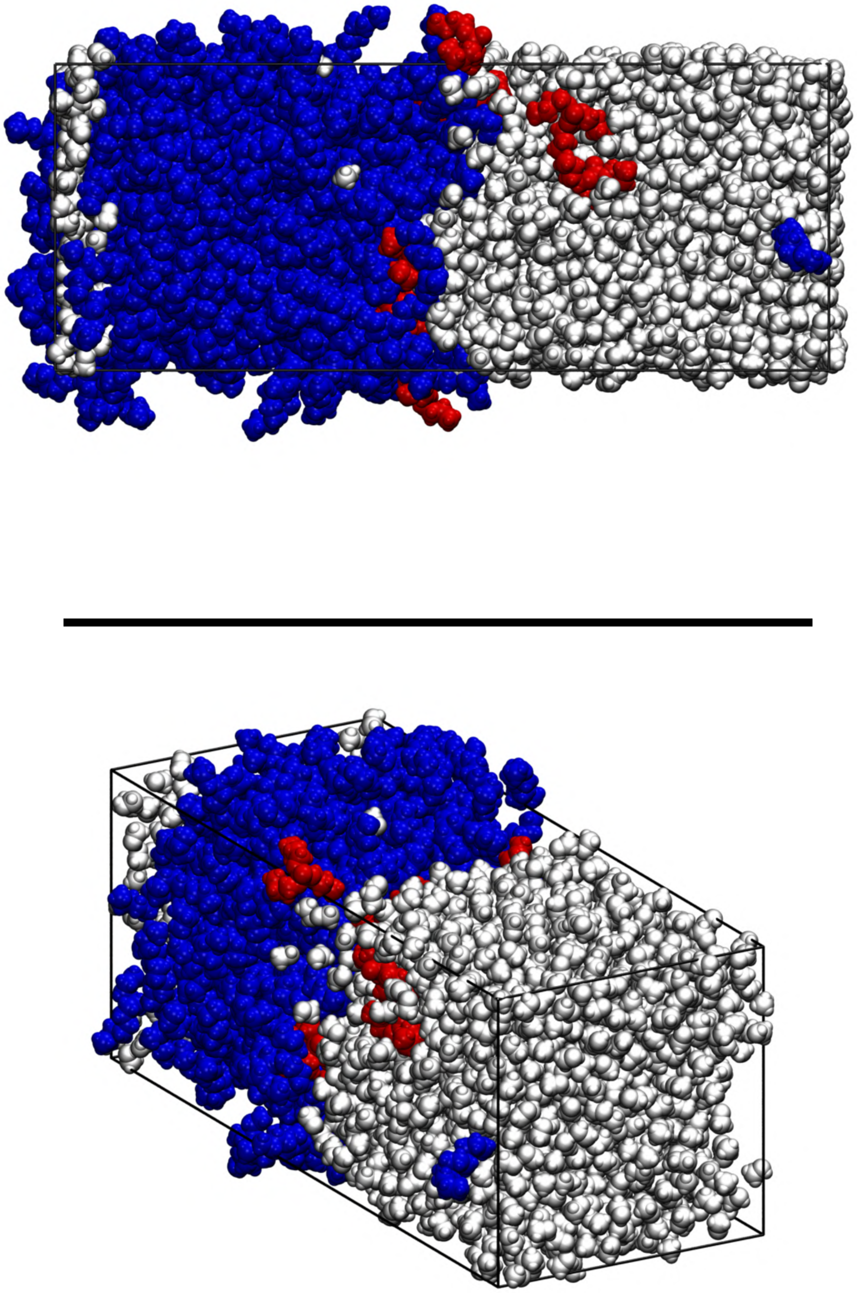

Figure S20: Snapshots of system CUB-MDT15. Atoms are represented by van der Waals spheres. White: MeOH, blue: DD, red: TBP.

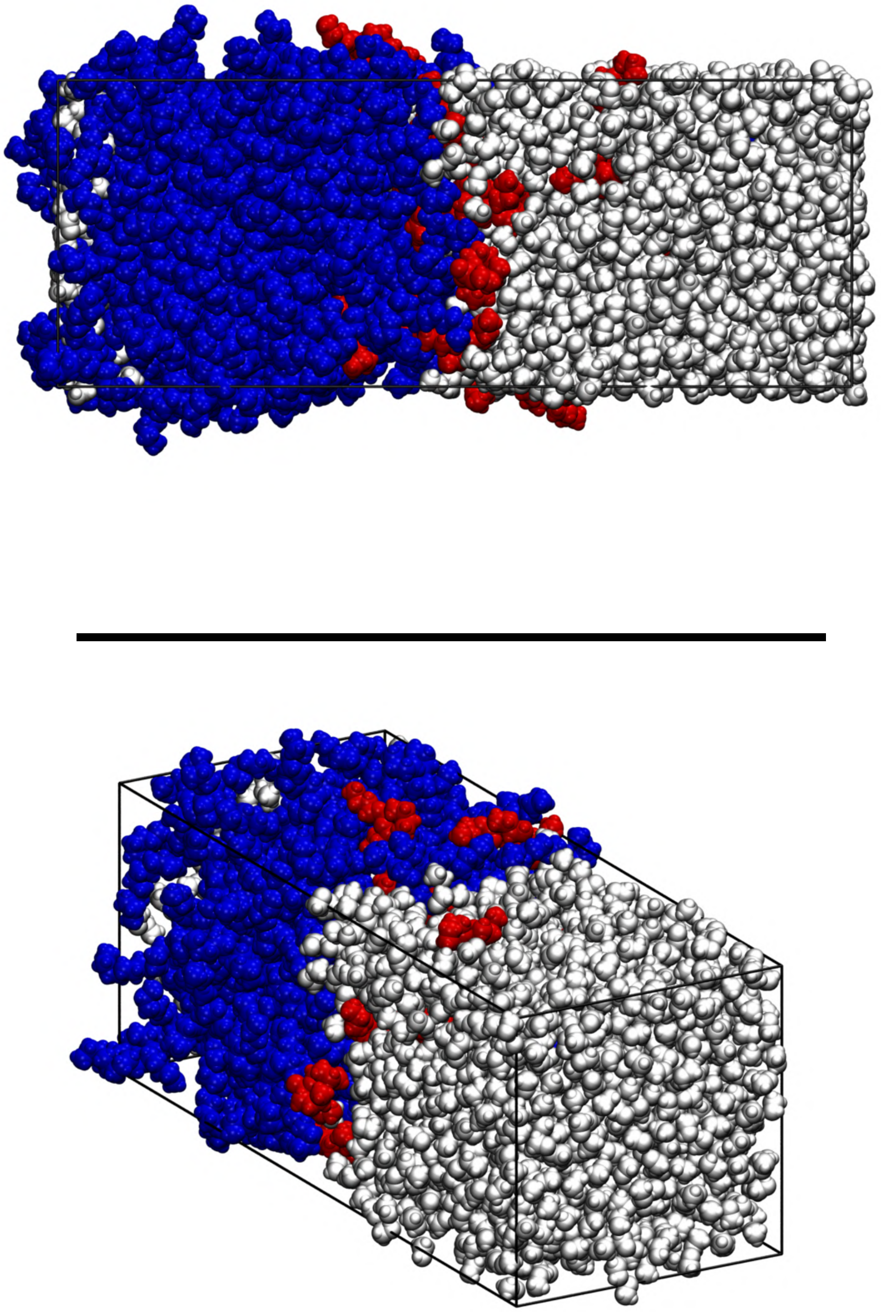

Figure S21: Snapshots of system CUB-MDT30. Atoms are represented by van der Waals spheres. White: MeOH, blue: DD, red: TBP.

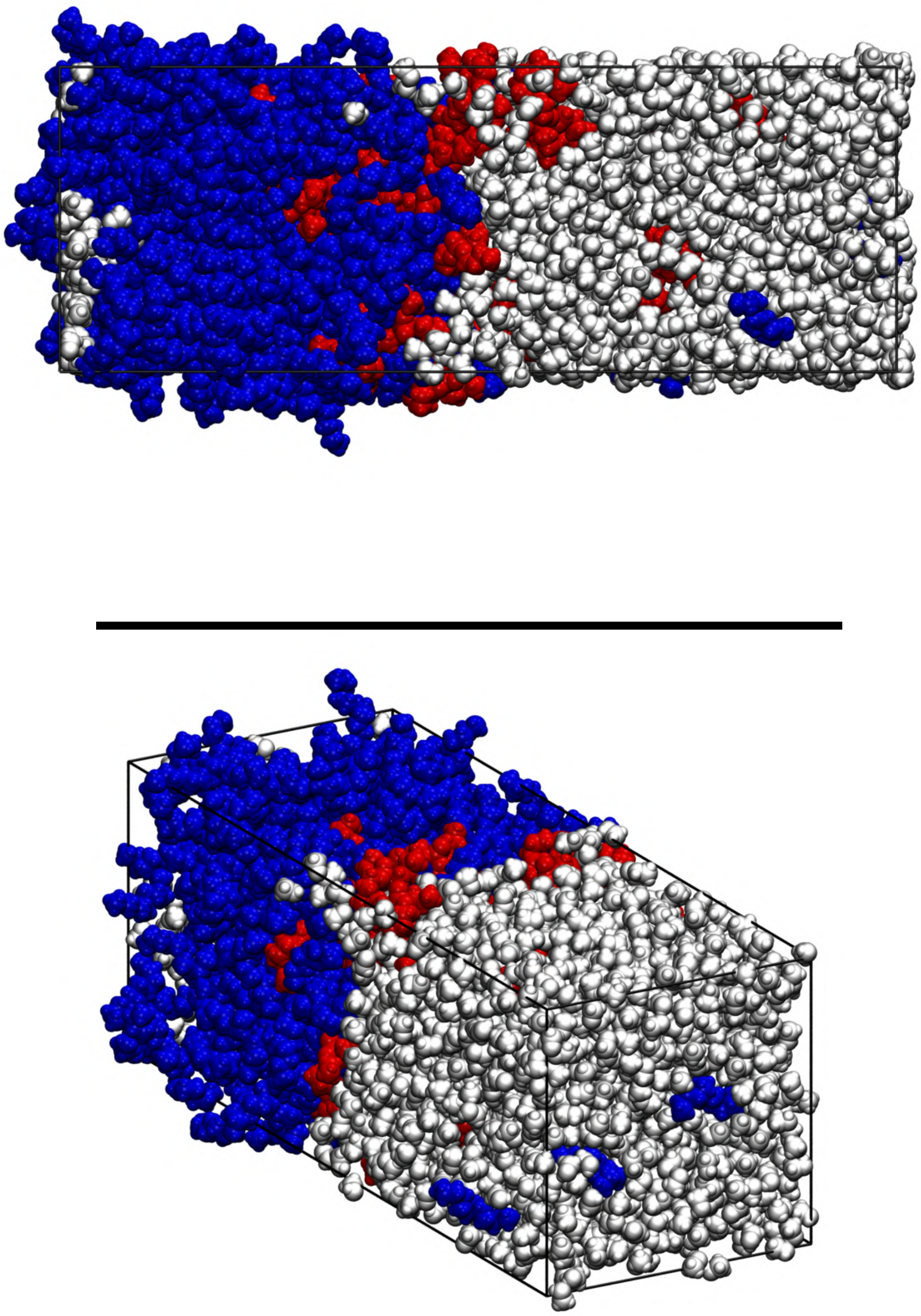

Figure S22: Snapshots of system CUB-MDT60. Atoms are represented by van der Waals spheres. White: MeOH, blue: DD, red: TBP.

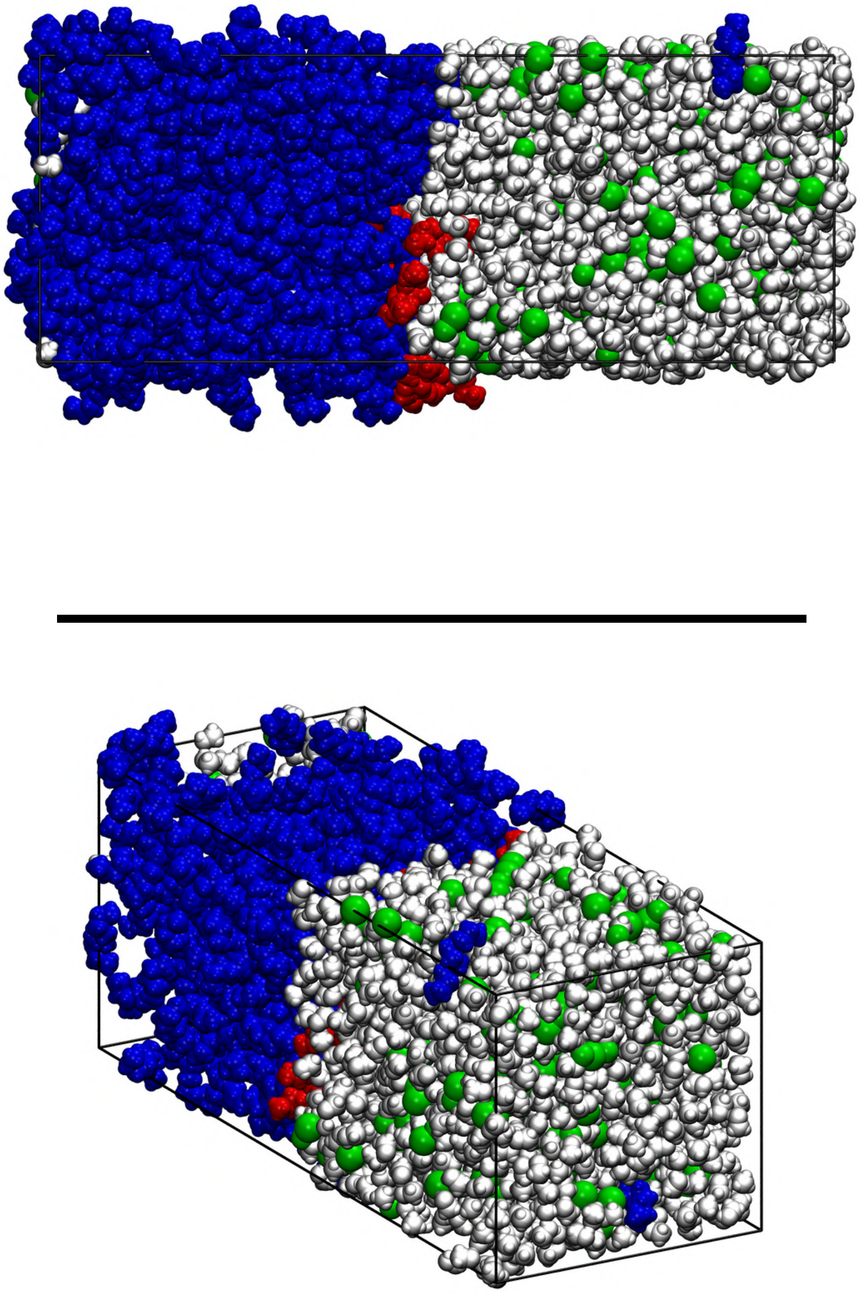

Figure S23: Snapshots of system CUB-MLDT15. Atoms are represented by van der Waals spheres. White: MeOH, blue: DD, red: TBP, green: LiCl.

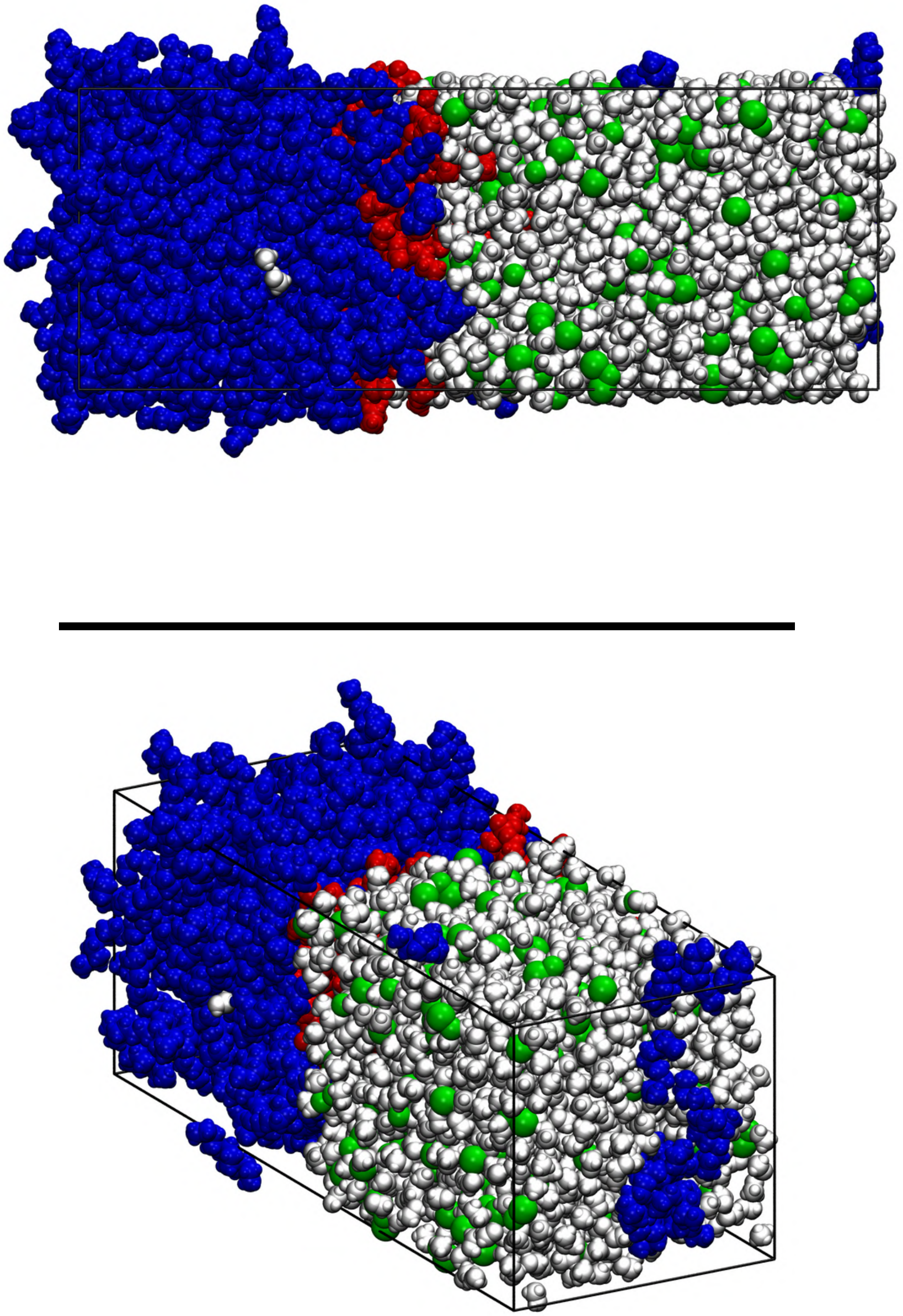

Figure S24: Snapshots of system CUB-MLDT30. Atoms are represented by van der Waals spheres. White: MeOH, blue: DD, red: TBP, green: LiCl.

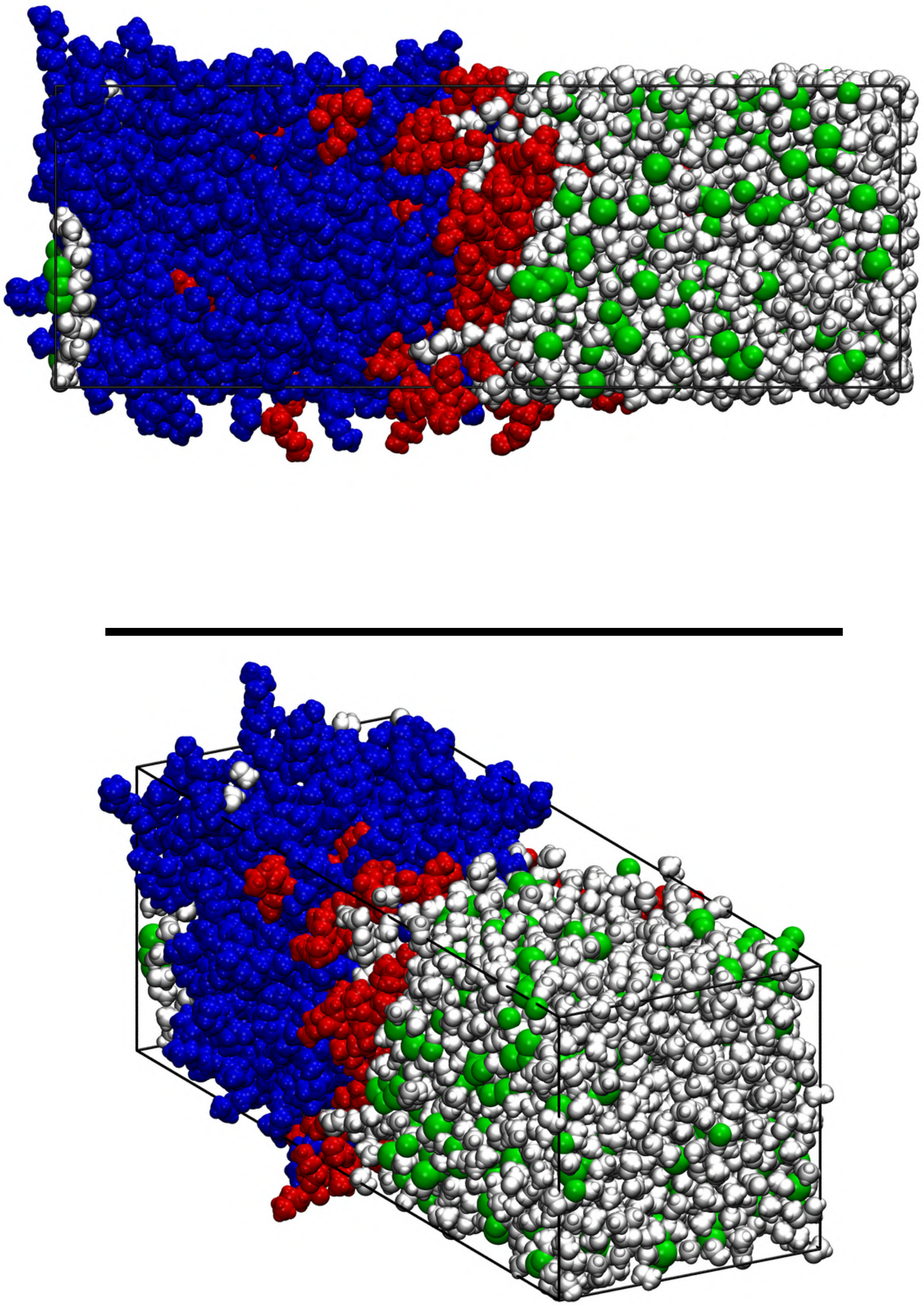

Figure S25: Snapshots of system CUB-MLDT60. Atoms are represented by van der Waals spheres. White: MeOH, blue: DD, red: TBP, green: LiCl.
